# Supplementary material for: UTAH: Using Telemedicine to improve early medical Abortion at Home: a protocol for a randomised controlled trial comparing face-to-face with telephone consultations for women seeking early medical abortion
Source: BMJ Open. 2021 Jun 16;11(6):e046628. doi: 10.1136/bmjopen-2020-046628 (PMC8211053; doi:10.1136/bmjopen-2020-046628)
Supplement: Supplementary data [file bmjopen-2020-046628supp001.pdf]

accord
Academic and Clinical Central Office for Research and Development

UTAH
PISCF 28 OCT 2019 v3.0
IRAS Project ID: 264265

Participant Information Sheet

UTAH: Using Telemedicine to improve early medical Abortion at Home

You are invited to take part in a research study. To help you decide whether or not to take part, it is important for you to understand why the research is being done and what it will involve. Please take time to read the following information carefully. Talk to others about the study if you wish. Contact us if there is anything that is not clear, or if you would like more information. Take time to decide whether or not you wish to take part.

What is the purpose of the study?

You are being asked to take part in a study comparing the use of telephone consultations to face-to-face consultations in the delivery of care for women thinking about having an early medical abortion at home.
Face-to-face consultations are currently the 'standard care' in NHS Lothian, however, in many services across the world, for example in parts of Canada and Australia, telephone consultations are used routinely and safely. Telephone consultations are also used by some UK services for women living at a distance from a clinic and can be convenient for women.
There has never been a study comparing telephone consultations to face-to-face consultations. The purpose of this study is to fill in that gap and find out if telephone consultations are as good as a face to face consultation for determining if medical abortion at home is suitable and for providing the information that a woman wishes about this. We also want to know if telephone consultations take more or less time (or the same time) as a face to face consultation and if women prefer them to a face-to-face consultation.

Why have I been invited to take part?

Women who intend to have a medical method of abortion at home and whose last menstrual period is less than 10 weeks prior to their appointment are eligible to take part. A total of 1222 women are being recruited to the study, with half (611) receiving the standard face-to-face consultation and half (611) receiving a telephone consultation before a clinic visit.

Do I have to take part?

No, it is up to you to decide whether or not to take part. If you do decide to take part you will be given this information sheet to keep and be asked to sign a consent form. If you decide to take part you are still free to withdraw at any time and without giving a reason. Deciding not to take part or withdrawing from the study will not affect the healthcare that you receive, or your legal rights.

Reynolds-Wright JJ, et al. BMJ Open 2021; 11:e046628. doi: 10.1136/bmjopen-2020-046628

What will happen if I take part?

When you call the clinic to make an appointment to discuss abortion, the receptionist on the telephone will give you a date and time to the attend the clinic, and will also invite you to take part in the study if you meet the eligibility criteria. If you agree to take part, you will receive a call back at a convenient time from a member of the research team, either a doctor or a nurse, who will explain the study in more detail and obtain your consent to participating over the phone.

You will then be randomly allocated by a computer to one group of the study, (you will know which group you are in): Telephone Group or Face-to-Face Group.

Telephone group: you will proceed to having your consultation on the telephone either immediately or at a later time, whichever is convenient for you. You will still attend the clinic at the date and time given to you in order to have all of the usual routine tests which include an ultrasound scan (to confirm how many weeks pregnant you are), have a blood test taken (for infections and for blood group) and a swab (that you take yourself) for infection. At this visit you would also be provided with the medical treatment to end the pregnancy, assuming that this is what you wish and that this is still appropriate for you based on the ultrasound scan of how many weeks pregnant you are at the clinic. Your chosen method of contraception can also be provided for you at this visit. If you change your mind, prefer a different method or based on the stage of the pregnancy a different method is indicated, you will be able to see a doctor or nurse in the clinic to discuss and plan this.

Face-to-face group: You will attend the clinic at the date and time given to you in order to have all of the usual routine tests (ultrasound scan, blood test and a swab). You will have the usual consultation with a doctor or nurse and proceed to treatment as usual. Your chosen method of contraception can also be provided for you at this visit. Participating in this arm of the study only involves completion of a short survey as described below.

For both groups, all of the tests and medical abortion treatments are the same.
For women in both groups a research nurse will ask you about your experience of the telephone or face to face consultation by a short telephone call interview (10 mins) two weeks after the treatment. If you prefer you can also choose to answer the same questions about your experience by completing a paper or online survey (whichever you prefer).

What are the possible benefits of taking part?

Direct benefits: If you are in the telephone group, you may spend less total time in the clinic, although it is also possible that you may spend the same time or longer than if you were in the face to face consultation group– this is one of the outcomes that the study is investigating. With the telephone consultation it is possible that you may have the consultation at a time and place that suits you better, minimising disruption to your daily life.

Indirect benefits: Your participation will help us to know how the two consultation options compare in terms of the outcome of the medical abortion, women’s acceptability of the consultations and how long the different consultations take. This will help inform us as to whether telephone consultations should be introduced as an option for women seeking a medical abortion at home in Scotland

What are the possible disadvantages of taking part?

If you are randomised to the telephone group, you will not be able to see the person you are speaking to and this may affect the satisfaction you have with the process. The content of the conversation and the verbal and written information that you receive will be the same.
If you are randomised to the face-to-face group, you do not face any additional risk or disadvantage compared to not participating in the study, as this is the usual standard care at the clinic. You will only have the inconvenience of completing the questionnaires that form part of the study, but these are brief and can be completed by the research nurse at a short telephone call (10 minutes).

What if there are any problems?

If you have a concern about any aspect of this study please contact [Removed for Publication], Research Nurse on [Telephone Number removed for publication], who will do their best to answer your questions.
In the unlikely event that something goes wrong and you are harmed during the research and this is due to someone's negligence then you may have grounds for a legal action for compensation against NHS Lothian but you may have to pay your legal costs. The normal National Health Service complaints mechanisms will still be available to you (if appropriate).

What will happen if I don't want to carry on with the study

You are free to withdraw from the study at any time, without this affecting your care at the clinic whatsoever. If you do withdraw from the study, any non-identifiable data already collected will be retained.

What happens when the study is finished?

When the study ends, identifiable data will be retained for 5 years in line with NHS Lothian Policy. Your data will be stored on an NHS Lothian Computer/Server. With your permission, some of your anonymised data will be kept for up to 10 years and may be used in future studies, but this information will not be directly linked to you and other researchers will not be able to identify you from it.

Will my taking part be kept confidential?

All information which is collected about you during the course of the research will be kept strictly confidential. Any published information will not contain any identifiable information. If during the study, you disclose information that means either you or another person (adult or child) may be at risk of harm, we will need to break confidentiality and inform the clinical team and any additional appropriate agencies as per the clinic's policy. If this happens, we will inform you at the time. For details on what data will be held about you and who will hold and store this information please refer to the Data Protection Information Sheet.

What will happen to the results of the study?

The results of the study will be published in a medical journal and presented at an international conference about reproductive health and contraception. Women who take

part in the study will not be able to be identified in any publication. If you wish, we can supply a summary of the findings to you via an email or postal address.

Who is organising and funding the research?

This study has been organised by the MRC Centre for Reproductive Health, University of Edinburgh and the Chalmers Centre for Sexual and Reproductive Health, NHS Lothian. Additionally, The Edinburgh Clinical Trials Unit (part of University of Edinburgh) will be providing database support, statistical and general trial management support. The study has been sponsored by ACCORD, a partnership between the University of Edinburgh and NHS Lothian.

The study is being funded by Edinburgh Family Planning Trust.

Who has reviewed the study?

The study proposal has been reviewed by NHS Lothian Research and Development.

The public have been involved in the development of this study, via review of the protocol and study documents by members of a community action group that support women in Edinburgh who receive abortion care.

All research in the NHS is looked at by an independent group of people called a Research Ethics Committee. A favourable ethical opinion has been obtained from South East Scotland REC 01. NHS Management Approval has also been given.

Researcher Contact Details

If you have any further questions about the study please contact [deleted for publication], Research Nurse on [telephone number] or email on: [email address]

Independent Contact Details

If you would like to discuss this study with someone independent of the study please contact [deleted for publication], Consultant Gynaecologist on [email address]

Complaints

If you wish to make a complaint about the study please contact:

[details removed for publication]

You can also do this through the NHS Complaints Procedure:
Patient Experience Team,
[details removed for publication]

accord
Academic and Clinical Central Office for Research and Development
NHS Lothian

UTAH
PISCF 28 OCT 2019 v3.0
IRAS Project ID 264265

Participant ID: **Participant name:**

RECORD OF VERBAL CONSENT

UTAH: Using Telemedicine to improve early medical Abortion at Home

Please **initial** box

1. I confirm that the participant has read and understood the information sheet (DD MMM YYYY and Version Number) and the Data Protection Information Sheet (DD MMM YYYY and Version Number) for the above study. They have had the opportunity to consider the information, ask questions and have had these questions answered satisfactorily. ☐
2. The participant understands that their participation is voluntary and that they are free to withdraw at any time, without giving any reason and without their medical care and/or legal rights being affected. ☐
3. The participant gives permission for the research team to access their medical records for the purposes of this research study. ☐
4. The participant understands that relevant sections of their medical notes and data collected during the study may be looked at by individuals from the Sponsor (University of Edinburgh and NHS Lothian), from regulatory authorities or from the NHS organisation where it is relevant to their taking part in this research. They give permission for these individuals to have access to their data and/or medical records. ☐
5. The participant gives permission for their personal information (including name, address, date of birth, telephone number and consent form) to be passed to the University of Edinburgh and Edinburgh Clinical Trials Unit for administration of the study. ☐
6. The participant understands that data collected about them during the study may be converted to anonymised data. ☐
7. The participant agrees to their anonymised data being used in future ethically approved studies. Yes ☐ No ☐
8. The participant agrees to take part in the above study. ☐

Name of Person Receiving Consent	Date	Signature

On the day of clinic attendance – please sign to confirm ongoing consent to participate and agreement with the above statements

Name of Participant	Date	Signature

1x original – into Site File; 1x copy – to Participant; 1x copy – into medical record

Reynolds-Wright JJ, *et al. BMJ Open* 2021; 11:e046628. doi: 10.1136/bmjopen-2020-046628
