# Supplementary material for: UTAH: Using Telemedicine to improve early medical Abortion at Home: a protocol for a randomised controlled trial comparing face-to-face with telephone consultations for women seeking early medical abortion
Source: BMJ Open. 2021 Jun 16;11(6):e046628. doi: 10.1136/bmjopen-2020-046628 (PMC8211053; doi:10.1136/bmjopen-2020-046628)
Supplement: Supplementary data [file bmjopen-2020-046628supp002.pdf]

UTAH – Questionnaire 1 STUDY NUMBER: __________

Name of interviewer____

Date of interview (dd/mm/yy)___________________________________

We would be grateful if you would spend some time filling out this questionnaire. It should take you about 10 minutes. The questionnaire asks about your experience of your recent consultation for medical abortion and your plans for contraception.

Please CIRCLE your response.

1. What kind of consultation did you receive?
    a. Face-to-face
    b. Telephone
    c. Telephone at first but then another consultation in clinic (NOTE: this means having a new consultation with a doctor, not just meeting the nurse to collect your medicines)
2. How acceptable did you find having your consultation this way?
    a. Very acceptable
    b. Somewhat acceptable
    c. Neutral
    d. Somewhat unacceptable
    e. Very unacceptable
3. How acceptable have you found the whole process so far?
    a. Very acceptable
    b. Somewhat acceptable
    c. Neutral
    d. Somewhat unacceptable
    e. Very unacceptable
4. Now that you have had your consultation, how well prepared do you feel?
    a. Very prepared
    b. Somewhat prepared
    c. Neutral
    d. Somewhat unprepared
    e. Very unprepared

UTAH – Questionnaire 1 STUDY NUMBER: __________

5. How satisfied with the consultation you had?
   a. Very satisfied
   b. Somewhat satisfied
   c. Neutral
   d. Somewhat unsatisfied
   e. Very unsatisfied
6. Do you feel that all of your questions were answered by the consultation?
   a. Yes
   b. I have some questions still
   c. No, none of my questions were answered
7. What did you think of duration of the consultation?
   a. Much too long
   b. A bit longer than I wanted
   c. Just right
   d. A bit shorter than I wanted
   e. Much too short
8. What time of day was your consultation?
   a. Morning (08.00 – 12.00)
   b. Afternoon (12.00-17.00)
   c. Evening (17.00-20.00)
9. How convenient was the time of day of your consultation?
   a. Very convenient
   b. Somewhat convenient
   c. Neutral
   d. Somewhat inconvenient
   e. Very inconvenient
10. What method of contraception (if any) are you planning to start following your abortion treatment?
   a. Combined hormonal contraceptive pill / patch or ring
   b. Progestogen only pill (mini pill)
   c. Male condom
   d. Contraceptive injection’ jag’ (Depo Provera or Sayana)
   e. Implant (Nexplanon)
   f. Copper Coil/intra-uterine device (IUD)

UTAH – Questionnaire 1 STUDY NUMBER: __________

g. Intrauterine system (Mirena or Jaydess)
h. Female condom
i. Cap/diaphragm
j. Partner has been sterilised (vasectomy)
k. I have been sterilised
l. I am currently pregnant
m. Other method of protection-**please write here what this is**

   ____

n. I am not planning to use any method of contraception
