# Supplementary material for: UTAH: Using Telemedicine to improve early medical Abortion at Home: a protocol for a randomised controlled trial comparing face-to-face with telephone consultations for women seeking early medical abortion
Source: BMJ Open. 2021 Jun 16;11(6):e046628. doi: 10.1136/bmjopen-2020-046628 (PMC8211053; doi:10.1136/bmjopen-2020-046628)
Supplement: Supplementary data [file bmjopen-2020-046628supp003.pdf]

UTAH – Questionnaire 2 STUDY NUMBER: __________

We would be grateful if you would spend some time filling out this questionnaire. It should take you about 10 minutes. The questionnaire asks about your experience of your recent consultation for medical abortion, the result of your pregnancy test and if you chose a method of contraception.

Please CIRCLE responses.

1. What kind of consultation did you receive?
   a. Face-to-face
   b. Telephone
   c. Telephone at first but then another consultation in clinic (NOTE: this means having a new consultation with a doctor, not just meeting the nurse to collect your medicines)
2. Looking back, how acceptable did you find having your consultation this way?
   a. Very acceptable
   b. Somewhat acceptable
   c. Neutral
   d. Somewhat unacceptable
   e. Very unacceptable
3. Looking back, what did you think of duration of the consultation?
   a. Much too long
   b. A bit longer than I wanted
   c. Just right
   d. A bit shorter than I wanted
   e. Much too short
4. Looking back, how well prepared were you?
   a. Very prepared
   b. Somewhat prepared
   c. Neutral
   d. Somewhat unprepared
   e. Very unprepared
5. If you had a good friend who was thinking about having an abortion knowing what you know now, would you recommend the same type of consultation?
   a. Yes
   b. No
   c. Not sure

UTAH – Questionnaire 2 STUDY NUMBER: __________

6. Did you start the method of contraception you left clinic with?
   a. Yes
   b. No – I have since chosen a different method
   c. No – I have not started but I am waiting for a coil/IUD/IUS
   d. No – I did not leave with a method
7. If you were to design the perfect service, which of the following would be important to you:

	Very Unimportant	Somewhat Unimportant	Neutral	Somewhat Important	Very Important
Evening face-to-face clinic					
Evening telephone consultation					
Skype or video consultation					
A mobile phone app to send/receive information in advance					
Online booking					
Medication posted to me					
Medication that could collect from a local pharmacy					
Able to get the treatment from my GP					
