# Supplementary material for: UTAH: Using Telemedicine to improve early medical Abortion at Home: a protocol for a randomised controlled trial comparing face-to-face with telephone consultations for women seeking early medical abortion
Source: BMJ Open. 2021 Jun 16;11(6):e046628. doi: 10.1136/bmjopen-2020-046628 (PMC8211053; doi:10.1136/bmjopen-2020-046628)
Supplement: Author's manuscript [file bmjopen-2020-046628.draft_revisions.pdf]

BMJ Open

BMJ Open is committed to open peer review. As part of this commitment we make the peer review history of every article we publish publicly available.

When an article is published we post the peer reviewers' comments and the authors' responses online. We also post the versions of the paper that were used during peer review. These are the versions that the peer review comments apply to.

The versions of the paper that follow are the versions that were submitted during the peer review process. They are not the versions of record or the final published versions. They should not be cited or distributed as the published version of this manuscript.

BMJ Open is an open access journal and the full, final, typeset and author-corrected version of record of the manuscript is available on our site with no access controls, subscription charges or pay-per-view fees (http://bmjopen.bmj.com).

If you have any questions on BMJ Open's open peer review process please email info.bmjopen@bmj.com

BMJ Open

# UTAH: Using Telemedicine to improve early medical Abortion at Home – a protocol for a randomised controlled trial comparing face-to-face with telephone consultations for women seeking early medical abortion.

| | |
|---|---|
| Journal: | *BMJ Open* |
| Manuscript ID | bmjopen-2020-046628 |
| Article Type: | Protocol |
| Date Submitted by the Author: | 05-Nov-2020 |
| Complete List of Authors: | Reynolds-Wright, John; The University of Edinburgh MRC Centre for Reproductive Health, The Queen's Medical Research Institute; NHS Lothian, Chalmers Centre for Sexual and Reproductive Health<br>Norrie, John; Edinburgh Clinical Trials Unit, University of Edinburgh No. 9, Bioquarter<br>Cameron, Sharon; University of Edinburgh, Obstetrics and Gynaecology; NHS Lothian, Sexual and Reproductive Health |
| Keywords: | GYNAECOLOGY, Reproductive medicine < GYNAECOLOGY, Community gynaecology < GYNAECOLOGY |
| | |

SCHOLARONE™
Manuscripts

*I, the Submitting Author has the right to grant and does grant on behalf of all authors of the Work (as defined in the below author licence), an exclusive licence and/or a non-exclusive licence for contributions from authors who are: i) UK Crown employees; ii) where BMJ has agreed a CC-BY licence shall apply, and/or iii) in accordance with the terms applicable for US Federal Government officers or employees acting as part of their official duties; on a worldwide, perpetual, irrevocable, royalty-free basis to BMJ Publishing Group Ltd ("BMJ") its licensees and where the relevant Journal is co-owned by BMJ to the co-owners of the Journal, to publish the Work in this journal and any other BMJ products and to exploit all rights, as set out in our licence.*

*The Submitting Author accepts and understands that any supply made under these terms is made by BMJ to the Submitting Author unless you are acting as an employee on behalf of your employer or a postgraduate student of an affiliated institution which is paying any applicable article publishing charge ("APC") for Open Access articles. Where the Submitting Author wishes to make the Work available on an Open Access basis (and intends to pay the relevant APC), the terms of reuse of such Open Access shall be governed by a Creative Commons licence – details of these licences and which Creative Commons licence will apply to this Work are set out in our licence referred to above.*

*Other than as permitted in any relevant BMJ Author's Self Archiving Policies, I confirm this Work has not been accepted for publication elsewhere, is not being considered for publication elsewhere and does not duplicate material already published. I confirm all authors consent to publication of this Work and authorise the granting of this licence.*

For peer review only

## Title

UTAH: Using Telemedicine to improve early medical Abortion at Home – a protocol for a randomised controlled trial comparing face-to-face with telephone consultations for women seeking early medical abortion.


## Authors

John J Reynolds-Wright[1,2] email: jjrw@doctors.org.uk
John Norrie[3] email: j.norrie@ed.ac.uk
Sharon Cameron[1,2] email: Sharon.cameron@ed.ac.uk

## Affiliations

[1]MRC Centre for Reproductive Health, University of Edinburgh, Edinburgh, UK
[2]Chalmers Centre, NHS Lothian, Edinburgh, UK
[3]Edinburgh Clinical Trial Unit, University of Edinburgh, Edinburgh, UK

## Corresponding Author:

John J Reynolds-Wright
MRC Centre for Reproductive Health
The Queen's Medical Research Institute
Edinburgh BioQuarter
47 Little France Crescent
Edinburgh
EH16 4TJ
email: jjrw@doctors.org.uk


Word Count: 2732

Key Words: Gynaecology, Reproductive medicine, Community Gynaecology

For peer review only

## Abstract

### Introduction

Early medical abortion (EMA) is a two-stage process of terminating pregnancy using oral mifepristone (a progesterone-receptor antagonist) followed usually 1-2 days later by sublingual, vaginal or buccal misoprostol (a prostaglandin analogue). There are no published randomised controlled trials on the use of telemedicine for early medical abortion (EMA). Our proposed research will determine if telephone consultations for EMA (the commonest method of abortion in the UK) is non-inferior to standard face-to-face consultations with regards to efficacy of EMA.

### Methods and analysis

This study will be conducted as a randomised controlled trial (RCT). The recruitment target is 1222 participants and has been calculated using a binary outcome non-inferiority calculator with 90% power, one-sided 5% level of significance, 3% non-inferiority limit, 1:1 allocation and 10% compensation for loss to follow up.
The primary outcome is success of EMA (complete abortion rate). This will be determined, based upon a negative low-sensitivity urine pregnancy test result (2 weeks days after misoprostol use), and absence of surgical intervention (within 30 days of misoprostol) and absence of a diagnosis of ongoing pregnancy.
The main analysis will be a modified intention to treat (mITT) analysis. This will include all randomised women using EMA, with a non-pathological pregnancy, and follow-up for the main outcome. The study initiated on 13th January 2020 and is anticipated to finish July 2021.

### Ethics and dissemination

Ethical approval was given by the South East Scotland NHS Research Ethics Committee, reference: 19/SS/0111. Results will be published in peer-reviewed journals, presented at clinical and academic meetings, and shared with participants via the clinic website.

### Registration details

UTAH was registered with clinicaltrials.gov on 25th October 2019. Unique identifier: NCT04139382.


### Strengths and limitations of this study

- This is the first study devised as a randomised-controlled trial to evaluate the safety and efficacy of telemedicine in early medical abortion.
- The study is design is aligned with the CONSORT, SPIRIT and Medical Abortion Reporting of Efficacy (MARE) recommendations in order to maximize the rigor and quality of the trial.

For peer review only

- The large sample size will allow a statistically and clinically meaningful analysis of the results.
- Due to the medico-legal framework surrounding abortion, the telemedicine arm of the study still needs to attend in person to receive medications.
- The primary outcome relies on participant report of low sensitivity urine pregnancy test result.

For peer review only

# Introduction

We plan a trial comparing telephone consultations for women requesting early medical abortion (EMA - under 10 weeks pregnant) to regular face-to-face consultations. In Scotland in 2018, 7 out of 10 women having an abortion chose EMA (1). In many settings, including Scotland, the clinic visit for a consultation to discuss a request for EMA is lengthy. Women can struggle with time off work or childcare for daytime appointments. There is evidence from observational studies that telephone consultations for EMA may be a safe and acceptable alternative(2-6). In our study, women seeking EMA will be randomised to face-to-face (standard care) or a planned telephone consultation (in advance of the clinic visit). We will determine the success of EMA in both groups, women's satisfaction with the consultation and possible advantages and disadvantages of the telephone consultation. If the study shows that success of EMA is maintained with a telephone consultation and that this model is acceptable to women, then this may change EMA provision throughout Scotland and other countries.

**Background**

Abortion care is common, with approximately 1 in 3 women experiencing abortion in their lifetime. Each year approximately 200,000 abortions are performed in the UK and around 13,000 of these are in Scotland. 99% of abortions are delivered by the NHS in Scotland (1), compared to England and Wales, where 70% are delivered outside the NHS by the independent sector (8). Furthermore, Scotland has higher uptake of medical methods of abortion compared to England and Wales. In Scotland in 2017, 80% of all abortions were conducted in early pregnancy (under 10 weeks) and over 90% of these were medical abortions - early medical abortion (EMA) (1). The World Health Organization recommend that women can reliably self-manage much of EMA with support from a clinician (9).

In Scotland, women who wish an EMA, typically make a single visit to a clinic for a consultation and for assessment of gestation, receipt of mifepristone and misoprostol (to self-administer at home), receipt of contraception and instructions on how to self-assess the success of the abortion (using a self-performed urinary pregnancy test) (10,11,12). This clinic visit can last 2-3 hours; much of which may be time spent in the waiting room, waiting to be seen. Moreover, a significant proportion of consultation time is standard history taking and information giving and could be delivered via the telephone, an app or video call rather than face-to-face. Telephone consultations could add flexibility for women (e.g. consultation in the evening), reduce the footfall in clinics (shorter time spent in clinic) and allow for more flexible staff working (office working, evening working, etc). There is observational evidence from other countries where abortion is legal to support use of telemedicine/telephone consultations for assessment of EMA (2-6). It is also possible that the consultation in advance of a clinic visit (for confirmation of decision, ultrasound and to collect medications) could mean that the subsequent clinic consultation is shorter with possible efficiencies for the service, such as more effective use of medical staffing. Additionally, if women are better informed about EMA in advance of the clinic, the clinic consultation may be better utilised to answer outstanding questions that the woman may have or discuss and provide ongoing contraception. Indeed, there is some observational evidence that telephone

For peer review only

counselling may be associated with higher uptake of post abortion contraception (7). This could translate into fewer subsequent unintended pregnancies for women. Around 2400 abortions take place in NHS Lothian annually (1) and most women (80%) women attend a community abortion service at Chalmers Centre. In 2018, over 70% of abortions in this service were EMA (13).
We wish to determine if telephone consultations for assessment of women who are potentially eligible for EMA are non-inferior to face-to-face consultations (in terms of successful outcome of EMA). We designed a study of a telephone consultation assessment service via a randomised controlled trial (RCT). This has not been conducted before. This will be conducted within the framework of the 1967 Abortion Act (14). This RCT will provide robust data to support future service development nationally. Telephone consultations may make abortion services more accessible for women (especially those with work or child care commitments and vulnerable women), possibly more efficient and affords the possibility of future cover for 'remote' services at other sites or health boards, as women could have an ultrasound for gestational dating and any other tests locally, but with consultations delivered by telephone. The aims of this study are in line with current Scottish Government policy on realistic medicine and on greater use of telemedicine services (15). Our Patient and Public Involvement team have helped develop this protocol and will continue to be involved throughout.

## Rationale for Study

There are no published randomised controlled trials on use of telemedicine or telephone abortion for EMA. The existing evidence base is observational and exists outside of the NHS healthcare framework and outside of the UK medicolegal framework. There are only 5 studies that report outcomes of EMA that have been conducted in settings where abortion is legal (USA, Canada, Australia) with much heterogeneity (2-6). Our proposed research has the potential to confirm that telephone consultations for EMA (the commonest method of abortion in Scotland) is non-inferior to standard face-to-face consultation with regards to efficacy. Efficacy of EMA has been chosen as we hypothesise that inferior consultations would have an impact on women's ability to self-manage EMA. This RCT will gather robust data regarding success of EMA, duration of clinic and telephone consultations, women's satisfaction with consultation (using validated questionnaires) and uptake of effective contraception post abortion. These findings can be used to inform service development and abortion care strategy at a national level in Scotland and elsewhere. The primary research question is 'Is a telephone consultation for EMA non-inferior to a face-to-face consultation?' The secondary research questions are: 'How do the consultations compare with regard to patient satisfaction, time taken, and uptake of effective methods of contraception?'

For peer review only

# Methods and analysis

## Study Design

This study will be conducted as a randomised controlled trial (RCT) to compare telephone with face-to-face consultations for women considering EMA at home.

### Primary Objective

To determine if EMA conducted following a telephone consultation is as effective (complete abortion rate) as following face to face consultation

### Secondary Objectives

To determine if a telephone consultation for EMA is associated with less total time spent at a clinic appointment to receive EMA, preparedness for EMA, level of satisfaction with consultation and effective contraception uptake compared to when women attend for a face-to-face consultation.

### Primary Endpoint

Success of EMA as defined by complete abortion rate. This will be determined, based upon self-reported negative low-sensitivity urine pregnancy test result (2 weeks after misoprostol) and review of clinical database at 6 weeks to confirm final outcome of pregnancy.

### Secondary Endpoints

Women's reported 'preparedness' for EMA as assessed by pre-abortion questionnaire, when they collect their pack of medications.
Satisfaction with consultation type as assessed by post-consultation questionnaire, conducted by telephone at 2 weeks.
Uptake of effective contraception after EMA as assessed by case note review.
Proportion of patients that are ineligible for EMA following ultrasound scan
Total time spent in clinic (both telephone and face-to-face groups) and time taken for telephone consultation
Unscheduled contact with abortion service or hospital within 4 weeks of EMA for concern related to EMA.

## Study Population

A total of 1222 participants randomised to receive telephone consultation (n=611) or face-to-face (n=611).
The success of EMA (primary outcome) is assumed as 97%. The recruitment target has been calculated using a binary outcome non-inferiority calculator with 90% power, one-sided 5% level of significance, 3% non-inferiority limit, 1:1 allocation and 10% compensation for loss to follow up (16). This will give us an adequately powered sample that will show statistical significance in efficacy findings.

For peer review only

The NHS Lothian abortion service cares for approximately 2400 women each year and of those 70% would be eligible to participate in the study. Over 18 months we should achieve adequate recruitment even if 50% of potential participants decline to participate.

### Inclusion Criteria

Self-reported last menstrual period (LMP) less than 10 weeks on day of appointment
Self-referral to Lothian Abortion Referral Service (LARS)
Aged 16 or over at the time of procedure
Preference for EMA
Ability to give informed consent

### Exclusion Criteria

Requires interpreter
Patient preference for surgical method of abortion

## Identifying Participants

The administrative staff of LARS will collect the usual details from women who self-refer for abortion (by telephone) and give them the next available date for the clinic so that participants in both study arms will receive an ultrasound scan, blood tests and sexual health screening as per usual care.
For women who meet the inclusion criteria, administrative staff will then read a short script about the study. If women express interest in participating, then permission will be sought for the research doctor or nurse to contact them by telephone at a convenient time to woman to discuss study participation. Interested women will also be directed to the clinic website where they can read the Participant Information Sheet (PIS) and consent form in advance of the call from the research doctor or nurse.

## Consent

Consent will be obtained from participants by the research doctor or nurse verbally over the telephone using a standard form. The participant will then be randomised to receive either a telephone consultation or a face-to-face consultation. When participants arrive in clinic, they will be asked to sign an affirmation that they continue to consent in the project.
The Participant Information Sheet and Consent Form are available as Supplementary File Appendix 1.
Randomisation lists will be generated by the Edinburgh Clinical Trials Unit (ECTU) and randomisation is performed by research staff using REDCap (Research Electronic Data Capture) software hosted at University of Edinburgh (17,18).

## Withdrawal of Participants

Participants are free to withdraw from the study at any point or a participant can be withdrawn by the Investigator. If withdrawal occurs, the primary reason for withdrawal will be documented in the participant’s case report form, if possible. The participant will have the option of withdrawal from all aspects of the trial but

For peer review only

continued use of data collected up to that point. To safeguard rights, the minimum personally identifiable information possible will be collected.

## Study Assessments

| Assessment | When | Administered by | Description | Study Arm |
|---|---|---|---|---|
| Consultation duration | During telephone consultation/face-to-face consultation | Research Doctor or Nurse | Duration of face-to-face/telephone assessment consultation plus time spent in clinic on day of attendance. | Both arms |
| Questionnaire 1 | At the abortion clinic, following consultation prior to commencing abortion | Research Doctor or Nurse | A researcher-administered questionnaire identifying how prepared participant feels for EMA, how satisfied they were with consultation, and plans for contraception following EMA. Demographic information will also be collected at this point. | Both arms |
| Questionnaire 2 | Over the telephone/online/by post 14-20 days following EMA | Research Nurse or Doctor or self. | A researcher administered questionnaire to assess outcome of abortion by self-reported LSUPT outcome, satisfaction with whole abortion process and contraceptive outcome. | Both arms |

*Table 1. Study Assessments*

Study assessments are detailed in Table 1. There is no long term follow up. Participants are followed up at two weeks post abortion only.

## Data Collection

Baseline demographics: demographics, reproductive history and gestational age (based on ultrasound) will be collected on all participants.
Consultation time: duration of telephone consultation (minutes) and duration of face to face clinic consultation (minutes), total time spent in clinic on day of attendance for assessment (minutes)

Participant preparedness questionnaire: At clinic on first attendance – research nurse or doctor administered questionnaire to assess how prepared they feel.

For peer review only

Participant acceptability questionnaire:  At two weeks post abortion - research nurse administered telephone questionnaire using validated questions on acceptability of consultation. Alternatively, this can be self-completed online or a paper postal questionnaire (if participant prefers this mode).

Outcome of abortion: self-reported outcome of routine low sensitivity urine pregnancy test at 2 weeks, plus review of clinical database at 6 weeks to confirm final outcome of pregnancy.

Unscheduled contact (in person or telephone) with abortion service or hospital for concern related to EMA (clinical records review at six weeks)

**Data Management**

Personal Data

The following personal data will be collected as part of the research, we note that this data is already routinely collected in clinical practice as part of clinical history:

Name

Post code

Weight, height, BMI

Previous pregnancy history

Physical personal data will be stored by the research team at Chalmers Centre, NHS Lothian, in the research office, behind a locked door that requires an ID badge to access and inside a locked cabinet in the room.

Electronic personal data will be kept on an NHS Lothian shared drive in password protected files. Passwords will be kept by research team and a hard copy with the locked physical data.

Identifiable personal data will be stored for a maximum of 5 years. Totally de-identified data will be retained for 10 years in total.

Data will be shared with colleagues at the University of Edinburgh Clinical Trials Unit (ECTU) who will assist with database management and statistical support.

## Transfer of Data

Data collected or generated by the study (including personal data) will not be transferred to any external individuals or organisations outside of the Sponsoring organisations.

## Data Controller

The University of Edinburgh and NHS Lothian are joint data controllers.

## Data Breaches

Any data breaches will be reported to the University of Edinburgh and NHS Lothian Data Protection Officers who will onward report to the relevant authority according to the appropriate timelines if required.

For peer review only

## Statistics and Data Analysis

### Proposed analyses

Statistical analysis will be conducted in partnership with the Edinburgh Clinical Trials Unit, University of Edinburgh.

Descriptive statistics will be used to characterize the groups of individuals recruited to the trial to investigate comparability of the two groups at baseline.

For the primary outcome of efficacy of EMA, the main analysis will be a modified intention to treat (mITT) analysis. This will be all randomised women, with medical abortion, non-pathological pregnancy, and follow up/outcome for main outcome recorded within 6 weeks of the abortion treatment.

A sensitivity analysis will be performed on a strict ITT-population consisting of all randomised women having had medical abortion with non-pathological pregnancy. In this analysis it is required to impute the outcome for women lost to follow up.

Secondary outcomes will be analysed using appropriate tests depending upon the normality of the data. Results will be considered statistically significant if P-value <0.05.

No interim analysis is planned.

## Patient and Public Involvement

We consulted Abortion Rights Edinburgh, a local abortion and women's rights activism group. They kindly provided feedback on the trial rationale, study design and study protocol prior to submission for ethical approval. They have agreed to disseminate the trial findings to their membership and via their networks.

## Ethics and dissemination

### Ethical approval

Ethical approval has been granted by South East Scotland NHS Research Ethics Committee on 28th October 2019, reference: 19/SS/0111.

### Dissemination plan

Results will be published in peer-reviewed journals, and as presentations at national and international meetings. All data will be reported in full. Participants will be able to access a summary of the trial results via the clinic website. Abortion Rights Edinburgh will disseminate to their membership and networks.

For peer review only

**Study Status**

The study opened to recruitment on 13th January 2020 and is temporarily paused due to service changes during covid-19.

**Administrative Details**

UTAH was registered with clinicaltrials.gov on 25th October 2019. Unique identifier: NCT04139382.
UTAH is jointly sponsored by the University of Edinburgh (UK) and NHS Lothian (UK) via the ACCORD partnership and assigned the identifier AC19076. Protocol Version: 1.0; Date 18th September 2019.
The sponsor reviewed the study design and gave research and development approval to the trial. They are not involved in the collection, management, analysis or interpretation of the data, nor will they be involved in any report writing.

The research team are: John Reynolds-Wright (Clinical Research Fellow), Anne Johnstone (Clinical Research Nurse), Karen McCabe (Clinical Research Midwife), Claire Nicol (Lead Nurse, Abortion Service) and Sharon Cameron (Principle and Chief Investigator).

Authors' contributions: JJRW and SC equally contributed to the design of the protocol. JN contributed to the statistical analysis and sampling sections of the protocol.

Funding statement: This work was supported by the Edinburgh Family Planning Trust grant number EFPT/2019/UTAH. The project was conducted from the MRC Centre for Reproductive Health, supported by the Medical Research Council (grant MR/N022556/1).

Competing interests statement: Professor John Norrie was Deputy Chair of the National Institute of Health Research (NIHR) Health Technology Assessment (HTA) General Funding Committee (2016-2019) and is currently Chair of the Medical Research Council (MRC)/NIHR Efficacy and Mechanisms Evaluation (EME) Funding Committee.

Professor Sharon Cameron has no competing interests to declare.

Dr John Reynolds-Wright has no competing interests to declare.

For peer review only

## References

1.Information Services Division (ISD) Scotland, NHS National Services Scotland. Termination of Pregnancy Statistics: year ending December 2018. http://www.isdscotland.org/Health-Topics/Sexual-Health/Abortions/ (accessed 07 March 2020)

2. Hyland P, Raymond EG, Chong E. A direct-to-patient telemedicine abortion service in Australia: Retrospective analysis of the first 18 months. Aust N Z J Obstet Gynaecol 2018;58(3):335-340.

3. Grossman D, Grindlay K. Safety of medical abortion provided through telemedicine compared with in person. Obstet Gynaecol 2017;130(4):778-782.

4. Grossman D, Grindlay K, Buchacker T, Lane K, Blanchard K. Effectiveness and acceptability of medical abortion provided through telemedicine. Obstet Gynecol 2011;118:296–303

5. Wiebe ER. Use of telemedicine for providing medical abortion. International journal of gynaecology and obstetrics: the official organ of the International Federation of Gynaecology and Obstetrics. 2014;124(2):177-8

6. Kohn JE, Snow JL, Simons HR, Seymour JW, Thompson T, Grossman D. Medication Abortion Provided Through Telemedicine in Four U.S. States Obstetrics & Gynecology: August 2019 - Volume 134 - Issue 2 - p 343-350 doi: 10.1097/AOG.0000000000003357

7. Lohr PA, Aiken ARA, Forsyth T, Trussell J. Telephone or integrated contraception counselling before abortion: impact on method choice and receipt. BMJ Sex Reprod Health. 2018; 44(2):114-121

8. Abortion Statistics for England and Wales 2018. https://www.gov.uk/government/statistics/abortion-statistics-for-england-and-wales-2018 (accessed 07 March 2020)

9. World Health Organization. Health worker roles in providing safe abortion care and post-abortion contraception. http://www.who.int/reproductivehealth/publications/unsafe_abortion/abortion-task-shifting/en/. (Accessed 07 March 2020)

10. Scottish Government. Chief Medical Officer Directorate. Abortion - Improvement to existing services - Approval for Misoprostol to be taken at home. https://www.sehd.scot.nhs.uk/cmo/CMO(2017)14.pdf (accessed 07 March 2020).

11. Cameron ST, Glasier A, Johnstone A, Dewart H, Campbell A. Can women determine the outcome of medical termination themselves? Contraception. 2015;91(1):6-11

For peer review only

12. Millar S, Cameron ST. Comparison of two low-sensitivity urine pregnancy tests for confirming the success of early medical abortion. BMJ Sex Reprod Health 2018;44(1):54-57

13. Finch R, McGeechan K, Johnstone A, Cameron S. Impact of the introduction of 'home' as a legal setting for self-administration of misoprostol for early medical abortions: a prospective observational cohort study. BMJ Sex Reprod Health 2019 Aug 17. pii: bmjsrh-2018-200278. doi: 10.1136/bmjsrh-2018-200278. [Epub ahead of print]

14. UK Government. The Abortion Act 1967. Accessed via: http://www.legislation.gov.uk/ukpga/1967/87 on 07 March 2020.

15. Calderwood C. Realising Realistic Medicine: Chief Medical Officer for Scotland annual report 2015-2016. Scottish Government. 27 February 2017. ISBN: 9781786526731. Accessible via: https://www.gov.scot/publications/chief-medical-officer-scotland-annual-report-2015-16-realising-realistic-9781786526731/pages/8/

16. Sealed Envelope Ltd. 2012. Power calculator for binary outcome non-inferiority trial. [Online] Available from: https://www.sealedenvelope.com/power/binary-noninferior/ [Accessed Sat Mar 07 2020]

17. PA Harris, R Taylor, R Thielke, J Payne, N Gonzalez, JG. Conde, Research electronic data capture (REDCap) – A metadata-driven methodology and workflow process for providing translational research informatics support, *J Biomed Inform. 2009 Apr;42(2):377-81.*

18. PA Harris, R Taylor, BL Minor, V Elliott, M Fernandez, L O'Neal, L McLeod, G Delacqua, F Delacqua, J Kirby, SN Duda, REDCap Consortium, The REDCap consortium: Building an international community of software partners, *J Biomed Inform. 2019 May 9 [doi: 10.1016/j.jbi.2019.103208]*

For peer review only

Academic and Clinical Central Office for Research and Development

UTAH
PISCF 28 OCT 2019 v3.0
IRAS Project ID: 264265

# Participant Information Sheet

## UTAH: Using Telemedicine to improve early medical Abortion at Home

**You are invited to take part in a research study. To help you decide whether or not to take part, it is important for you to understand why the research is being done and what it will involve. Please take time to read the following information carefully. Talk to others about the study if you wish. Contact us if there is anything that is not clear, or if you would like more information. Take time to decide whether or not you wish to take part.**

## What is the purpose of the study?

You are being asked to take part in a study comparing the use of telephone consultations to face-to-face consultations in the delivery of care for women thinking about having an early medical abortion at home.
Face-to-face consultations are currently the 'standard care' in NHS Lothian, however, in many services across the world, for example in parts of Canada and Australia, telephone consultations are used routinely and safely. Telephone consultations are also used by some UK services for women living at a distance from a clinic and can be convenient for women.
There has never been a study comparing telephone consultations to face-to-face consultations. The purpose of this study is to fill in that gap and find out if telephone consultations are as good as a face to face consultation for determining if medical abortion at home is suitable and for providing the information that a woman wishes about this. We also want to know if telephone consultations take more or less time (or the same time) as a face to face consultation and if women prefer them to a face-to-face consultation.

## Why have I been invited to take part?

Women who intend to have a medical method of abortion at home and whose last menstrual period is less than 10 weeks prior to their appointment are eligible to take part. A total of 1222 women are being recruited to the study, with half (611) receiving the standard face-to-face consultation and half (611) receiving a telephone consultation before a clinic visit.

## Do I have to take part?

No, it is up to you to decide whether or not to take part. If you do decide to take part you will be given this information sheet to keep and be asked to sign a consent form. If you decide to take part you are still free to withdraw at any time and without giving a reason. Deciding not to take part or withdrawing from the study will not affect the healthcare that you receive, or your legal rights.

For peer review only - http://bmjopen.bmj.com/site/about/guidelines.xhtml

## What will happen if I take part?

When you call the clinic to make an appointment to discuss abortion, the receptionist on the telephone will give you a date and time to the attend the clinic, and will also invite you to take part in the study if you meet the eligibility criteria. If you agree to take part, you will receive a call back at a convenient time from a member of the research team, either a doctor or a nurse, who will explain the study in more detail and obtain your consent to participating over the phone.

You will then be randomly allocated by a computer to one group of the study, (you will know which group you are in): Telephone Group or Face-to-Face Group.

Telephone group: you will proceed to having your consultation on the telephone either immediately or at a later time, whichever is convenient for you. You will still attend the clinic at the date and time given to you in order to have all of the usual routine tests which include an ultrasound scan (to confirm how many weeks pregnant you are), have a blood test taken (for infections and for blood group) and a swab (that you take yourself) for infection. At this visit you would also be provided with the medical treatment to end the pregnancy, assuming that this is what you wish and that this is still appropriate for you based on the ultrasound scan of how many weeks pregnant you are at the clinic. Your chosen method of contraception can also be provided for you at this visit. If you change your mind, prefer a different method or based on the stage of the pregnancy a different method is indicated, you will be able to see a doctor or nurse in the clinic to discuss and plan this.

Face-to-face group: You will attend the clinic at the date and time given to you in order to have all of the usual routine tests (ultrasound scan, blood test and a swab). You will have the usual consultation with a doctor or nurse and proceed to treatment as usual. Your chosen method of contraception can also be provided for you at this visit. Participating in this arm of the study only involves completion of a short survey as described below.

For both groups, all of the tests and medical abortion treatments are the same.
For women in both groups a research nurse will ask you about your experience of the telephone or face to face consultation by a short telephone call interview (10 mins) two weeks after the treatment. If you prefer you can also choose to answer the same questions about your experience by completing a paper or online survey (whichever you prefer).

## What are the possible benefits of taking part?

Direct benefits: If you are in the telephone group, you may spend less total time in the clinic, although it is also possible that you may spend the same time or longer than if you were in the face to face consultation group– this is one of the outcomes that the study is investigating. With the telephone consultation it is possible that you may have the consultation at a time and place that suits you better, minimising disruption to your daily life.

Indirect benefits: Your participation will help us to know how the two consultation options compare in terms of the outcome of the medical abortion, women's acceptability of the consultations and how long the different consultations take. This will help inform us as to whether telephone consultations should be introduced as an option for women seeking a medical abortion at home in Scotland

For peer review only - http://bmjopen.bmj.com/site/about/guidelines.xhtml

## What are the possible disadvantages of taking part?

If you are randomised to the telephone group, you will not be able to see the person you are speaking to and this may affect the satisfaction you have with the process. The content of the conversation and the verbal and written information that you receive will be the same.
If you are randomised to the face-to-face group, you do not face any additional risk or disadvantage compared to not participating in the study, as this is the usual standard care at the clinic. You will only have the inconvenience of completing the questionnaires that form part of the study, but these are brief and can be completed by the research nurse at a short telephone call (10 minutes).

## What if there are any problems?

If you have a concern about any aspect of this study please contact [Removed for Publication], Research Nurse on [Telephone Number removed for publication], who will do their best to answer your questions.
In the unlikely event that something goes wrong and you are harmed during the research and this is due to someone's negligence then you may have grounds for a legal action for compensation against NHS Lothian but you may have to pay your legal costs. The normal National Health Service complaints mechanisms will still be available to you (if appropriate).

## What will happen if I don't want to carry on with the study

You are free to withdraw from the study at any time, without this affecting your care at the clinic whatsoever. If you do withdraw from the study, any non-identifiable data already collected will be retained.

## What happens when the study is finished?

When the study ends, identifiable data will be retained for 5 years in line with NHS Lothian Policy. Your data will be stored on an NHS Lothian Computer/Server. With your permission, some of your anonymised data will be kept for up to 10 years and may be used in future studies, but this information will not be directly linked to you and other researchers will not be able to identify you from it.

## Will my taking part be kept confidential?

All information which is collected about you during the course of the research will be kept strictly confidential. Any published information will not contain any identifiable information. If during the study, you disclose information that means either you or another person (adult or child) may be at risk of harm, we will need to break confidentiality and inform the clinical team and any additional appropriate agencies as per the clinic's policy. If this happens, we will inform you at the time. For details on what data will be held about you and who will hold and store this information please refer to the Data Protection Information Sheet.

## What will happen to the results of the study?

The results of the study will be published in a medical journal and presented at an international conference about reproductive health and contraception. Women who take

For peer review only - http://bmjopen.bmj.com/site/about/guidelines.xhtml

part in the study will not be able to be identified in any publication. If you wish, we can supply a summary of the findings to you via an email or postal address.

## Who is organising and funding the research?

This study has been organised by the MRC Centre for Reproductive Health, University of Edinburgh and the Chalmers Centre for Sexual and Reproductive Health, NHS Lothian. Additionally, The Edinburgh Clinical Trials Unit (part of University of Edinburgh) will be providing database support, statistical and general trial management support. The study has been sponsored by ACCORD, a partnership between the University of Edinburgh and NHS Lothian.

The study is being funded by Edinburgh Family Planning Trust.

## Who has reviewed the study?

The study proposal has been reviewed by NHS Lothian Research and Development.

The public have been involved in the development of this study, via review of the protocol and study documents by members of a community action group that support women in Edinburgh who receive abortion care.

All research in the NHS is looked at by an independent group of people called a Research Ethics Committee. A favourable ethical opinion has been obtained from South East Scotland REC 01.  NHS Management Approval has also been given.

## Researcher Contact Details

If you have any further questions about the study please contact [deleted for publication], Research Nurse on [telephone number] or email on: [email address]

## Independent Contact Details

If you would like to discuss this study with someone independent of the study please contact [deleted for publication], Consultant Gynaecologist on [email address]

## Complaints

If you wish to make a complaint about the study please contact:

[details removed for publication]

You can also do this through the NHS Complaints Procedure:
Patient Experience Team,
[details removed for publication]

For peer review only - http://bmjopen.bmj.com/site/about/guidelines.xhtml

Academic and Clinical Central Office for Research and Development

UTAH
PISCF 28 OCT 2019 v3.0
IRAS Project ID 264265

**Participant ID:** **Participant name:**

# RECORD OF VERBAL CONSENT

## UTAH: Using Telemedicine to improve early medical Abortion at Home

Please **initial** box

1. I confirm that the participant has read and understood the information sheet (DD MMM YYYY and Version Number) and the Data Protection Information Sheet (DD MMM YYYY and Version Number) for the above study. They have had the opportunity to consider the information, ask questions and have had these questions answered satisfactorily. ☐
2. The participant understands that their participation is voluntary and that they are free to withdraw at any time, without giving any reason and without their medical care and/or legal rights being affected. ☐
3. The participant gives permission for the research team to access their medical records for the purposes of this research study. ☐
4. The participant understands that relevant sections of their medical notes and data collected during the study may be looked at by individuals from the Sponsor (University of Edinburgh and NHS Lothian), from regulatory authorities or from the NHS organisation where it is relevant to their taking part in this research. They give permission for these individuals to have access to their data and/or medical records. ☐
5. The participant gives permission for their personal information (including name, address, date of birth, telephone number and consent form) to be passed to the University of Edinburgh and Edinburgh Clinical Trials Unit for administration of the study. ☐
6. The participant understands that data collected about them during the study may be converted to anonymised data. ☐
7. The participant agrees to their anonymised data being used in future ethically approved studies. Yes ☐ No ☐
8. The participant agrees to take part in the above study. ☐

| Name of Person Receiving Consent | Date | Signature |
|---|---|---|

**On the day of clinic attendance – please sign to confirm ongoing consent to participate and agreement with the above statements**

| Name of Participant | Date | Signature |
|---|---|---|

1x original – into Site File; 1x copy – to Participant; 1x copy – into medical record

For peer review only - http://bmjopen.bmj.com/site/about/guidelines.xhtml

# Reporting checklist for protocol of a clinical trial.

Based on the SPIRIT guidelines.

## Instructions to authors

Complete this checklist by entering the page numbers from your manuscript where readers will find each of the items listed below.

Your article may not currently address all the items on the checklist. Please modify your text to include the missing information. If you are certain that an item does not apply, please write "n/a" and provide a short explanation.

Upload your completed checklist as an extra file when you submit to a journal.

In your methods section, say that you used the SPIRITreporting guidelines, and cite them as:

Chan A-W, Tetzlaff JM, Gøtzsche PC, Altman DG, Mann H, Berlin J, Dickersin K, Hróbjartsson A, Schulz KF, Parulekar WR, Krleža-Jerić K, Laupacis A, Moher D. SPIRIT 2013 Explanation and Elaboration: Guidance for protocols of clinical trials. BMJ. 2013;346:e7586

| | | Reporting Item | Page Number |
|---|---|---|---|
| **Administrative information** | | | |
| Title | #1 | Descriptive title identifying the study design, population, interventions, and, if applicable, trial acronym | 1 |
| Trial registration | #2a | Trial identifier and registry name. If not yet registered, name of intended registry | 2 |
| Trial registration: data set | #2b | All items from the World Health Organization Trial Registration Data Set | 2 |
| Protocol version | #3 | Date and version identifier | 9 |
| Funding | #4 | Sources and types of financial, material, and other support | 9 |
| Roles and responsibilities: | #5a | Names, affiliations, and roles of protocol contributors | 9 |

For peer review only

| | | | |
|---|---|---|---|
| contributorship | | | |
| Roles and responsibilities: sponsor contact information | #5b | Name and contact information for the trial sponsor | 9 |
| Roles and responsibilities: sponsor and funder | #5c | Role of study sponsor and funders, if any, in study design; collection, management, analysis, and interpretation of data; writing of the report; and the decision to submit the report for publication, including whether they will have ultimate authority over any of these activities | 9 |
| Roles and responsibilities: committees | #5d | Composition, roles, and responsibilities of the coordinating centre, steering committee, endpoint adjudication committee, data management team, and other individuals or groups overseeing the trial, if applicable (see Item 21a for data monitoring committee) | n/a |
| **Introduction** | | | |
| Background and rationale | #6a | Description of research question and justification for undertaking the trial, including summary of relevant studies (published and unpublished) examining benefits and harms for each intervention | 3 |
| Background and rationale: choice of comparators | #6b | Explanation for choice of comparators | 4 |
| Objectives | #7 | Specific objectives or hypotheses | 5 |
| Trial design | #8 | Description of trial design including type of trial (eg, parallel group, crossover, factorial, single group), allocation ratio, and framework (eg, superiority, equivalence, non-inferiority, exploratory) | 5 |
| **Methods: Participants, interventions, and outcomes** | | | |

For peer review only

| | | | |
|---|---|---|---|
| Study setting | #9 | Description of study settings (eg, community clinic, academic hospital) and list of countries where data will be collected. Reference to where list of study sites can be obtained | 5 |
| Eligibility criteria | #10 | Inclusion and exclusion criteria for participants. If applicable, eligibility criteria for study centres and individuals who will perform the interventions (eg, surgeons, psychotherapists) | 5,6 |
| Interventions: description | #11a | Interventions for each group with sufficient detail to allow replication, including how and when they will be administered | 5 |
| Interventions: modifications | #11b | Criteria for discontinuing or modifying allocated interventions for a given trial participant (eg, drug dose change in response to harms, participant request, or improving / worsening disease) | n/a |
| Interventions: adherance | #11c | Strategies to improve adherence to intervention protocols, and any procedures for monitoring adherence (eg, drug tablet return; laboratory tests) | n/a |
| Interventions: concomitant care | #11d | Relevant concomitant care and interventions that are permitted or prohibited during the trial | n/a |
| Outcomes | #12 | Primary, secondary, and other outcomes, including the specific measurement variable (eg, systolic blood pressure), analysis metric (eg, change from baseline, final value, time to event), method of aggregation (eg, median, proportion), and time point for each outcome. Explanation of the clinical relevance of chosen efficacy and harm outcomes is strongly recommended | 5 |
| Participant timeline | #13 | Time schedule of enrolment, interventions (including any run-ins and washouts), assessments, and visits for participants. A schematic diagram is highly recommended (see Figure) | 6/7 |
| Sample size | #14 | Estimated number of participants needed to achieve study objectives and how it was determined, including | 5 |

For peer review only

| | | | |
|---|---|---|---|
| | | clinical and statistical assumptions supporting any sample size calculations | |
| Recruitment | #15 | Strategies for achieving adequate participant enrolment to reach target sample size | 6 |
| **Methods: Assignment of interventions (for controlled trials)** | | | |
| Allocation: sequence generation | #16a | Method of generating the allocation sequence (eg, computer-generated random numbers), and list of any factors for stratification. To reduce predictability of a random sequence, details of any planned restriction (eg, blocking) should be provided in a separate document that is unavailable to those who enrol participants or assign interventions | 6 |
| Allocation concealment mechanism | #16b | Mechanism of implementing the allocation sequence (eg, central telephone; sequentially numbered, opaque, sealed envelopes), describing any steps to conceal the sequence until interventions are assigned | n/a |
| Allocation: implementation | #16c | Who will generate the allocation sequence, who will enrol participants, and who will assign participants to interventions | 6 |
| Blinding (masking) | #17a | Who will be blinded after assignment to interventions (eg, trial participants, care providers, outcome assessors, data analysts), and how | n/a |
| Blinding (masking): emergency unblinding | #17b | If blinded, circumstances under which unblinding is permissible, and procedure for revealing a participant's allocated intervention during the trial | n/a |
| **Methods: Data collection, management, and analysis** | | | |
| Data collection plan | #18a | Plans for assessment and collection of outcome, | 7 |

For peer review only

| | | | |
|---|---|---|---|
| | | baseline, and other trial data, including any related processes to promote data quality (eg, duplicate measurements, training of assessors) and a description of study instruments (eg, questionnaires, laboratory tests) along with their reliability and validity, if known. Reference to where data collection forms can be found, if not in the protocol | |
| Data collection plan: retention | #18b | Plans to promote participant retention and complete follow-up, including list of any outcome data to be collected for participants who discontinue or deviate from intervention protocols | 7 |
| Data management | #19 | Plans for data entry, coding, security, and storage, including any related processes to promote data quality (eg, double data entry; range checks for data values). Reference to where details of data management procedures can be found, if not in the protocol | 7 |
| Statistics: outcomes | #20a | Statistical methods for analysing primary and secondary outcomes. Reference to where other details of the statistical analysis plan can be found, if not in the protocol | 8 |
| Statistics: additional analyses | #20b | Methods for any additional analyses (eg, subgroup and adjusted analyses) | 8 |
| Statistics: analysis population and missing data | #20c | Definition of analysis population relating to protocol non-adherence (eg, as randomised analysis), and any statistical methods to handle missing data (eg, multiple imputation) | 8 |
| **Methods: Monitoring** | | | |
| Data monitoring: formal committee | #21a | Composition of data monitoring committee (DMC); summary of its role and reporting structure; statement of whether it is independent from the sponsor and competing interests; and reference to where further details about its charter can be found, if not in the protocol. Alternatively, an explanation of why a DMC is not needed | n/a |
| Data monitoring: | #21b | Description of any interim analyses and stopping | 8 |

For peer review only

| | | | |
|---|---|---|---|
| interim analysis | | guidelines, including who will have access to these interim results and make the final decision to terminate the trial | |
| Harms | #22 | Plans for collecting, assessing, reporting, and managing solicited and spontaneously reported adverse events and other unintended effects of trial interventions or trial conduct | n/a |
| Auditing | #23 | Frequency and procedures for auditing trial conduct, if any, and whether the process will be independent from investigators and the sponsor | n/a |
| **Ethics and dissemination** | | | |
| Research ethics approval | #24 | Plans for seeking research ethics committee / institutional review board (REC / IRB) approval | 9 |
| Protocol amendments | #25 | Plans for communicating important protocol modifications (eg, changes to eligibility criteria, outcomes, analyses) to relevant parties (eg, investigators, REC / IRBs, trial participants, trial registries, journals, regulators) | 9 |
| Consent or assent | #26a | Who will obtain informed consent or assent from potential trial participants or authorised surrogates, and how (see Item 32) | 6 |
| Consent or assent: ancillary studies | #26b | Additional consent provisions for collection and use of participant data and biological specimens in ancillary studies, if applicable | n/a |
| Confidentiality | #27 | How personal information about potential and enrolled participants will be collected, shared, and maintained in order to protect confidentiality before, during, and after the trial | 8 |
| Declaration of interests | #28 | Financial and other competing interests for principal investigators for the overall trial and each study site | 9 |
| Data access | #29 | Statement of who will have access to the final trial dataset, and disclosure of contractual agreements that limit such access for investigators | n/a |

For peer review only

| | | | |
|---|---|---|---|
| Ancillary and post trial care | #30 | Provisions, if any, for ancillary and post-trial care, and for compensation to those who suffer harm from trial participation | n/a |
| Dissemination policy: trial results | #31a | Plans for investigators and sponsor to communicate trial results to participants, healthcare professionals, the public, and other relevant groups (eg, via publication, reporting in results databases, or other data sharing arrangements), including any publication restrictions | 9 |
| Dissemination policy: authorship | #31b | Authorship eligibility guidelines and any intended use of professional writers | n/a |
| Dissemination policy: reproducible research | #31c | Plans, if any, for granting public access to the full protocol, participant-level dataset, and statistical code | n/a |
| **Appendices** | | | |
| Informed consent materials | #32 | Model consent form and other related documentation given to participants and authorised surrogates | appendix 1 |
| Biological specimens | #33 | Plans for collection, laboratory evaluation, and storage of biological specimens for genetic or molecular analysis in the current trial and for future use in ancillary studies, if applicable | n/a |

The SPIRIT checklist is distributed under the terms of the Creative Commons Attribution License CC-BY-ND 3.0. This checklist was completed on 04. November 2020 using https://www.goodreports.org/, a tool made by the EQUATOR Network in collaboration with Penelope.ai

For peer review only

BMJ Open

# UTAH: Using Telemedicine to improve early medical Abortion at Home – a protocol for a randomised controlled trial comparing face-to-face with telephone consultations for women seeking early medical abortion.


| | |
|---|---|
| Journal: | *BMJ Open* |
| Manuscript ID | bmjopen-2020-046628.R1 |
| Article Type: | Protocol |
| Date Submitted by the Author: | 25-Mar-2021 |
| Complete List of Authors: | Reynolds-Wright, John; The University of Edinburgh MRC Centre for Reproductive Health, The Queen's Medical Research Institute; NHS Lothian, Chalmers Centre for Sexual and Reproductive Health<br>Norrie, John; Edinburgh Clinical Trials Unit, University of Edinburgh No. 9, Bioquarter<br>Cameron, Sharon; University of Edinburgh, Obstetrics and Gynaecology; NHS Lothian, Sexual and Reproductive Health |
| <b>Primary Subject Heading</b>: | Sexual health |
| Secondary Subject Heading: | Health services research, Obstetrics and gynaecology, Patient-centred medicine, Reproductive medicine, Communication |
| Keywords: | GYNAECOLOGY, Reproductive medicine < GYNAECOLOGY, Community gynaecology < GYNAECOLOGY, Telemedicine < BIOTECHNOLOGY & BIOINFORMATICS, PUBLIC HEALTH, REPRODUCTIVE MEDICINE |
| | |


SCHOLARONE™
Manuscripts

*I, the Submitting Author has the right to grant and does grant on behalf of all authors of the Work (as defined in the below author licence), an exclusive licence and/or a non-exclusive licence for contributions from authors who are: i) UK Crown employees; ii) where BMJ has agreed a CC-BY licence shall apply, and/or iii) in accordance with the terms applicable for US Federal Government officers or employees acting as part of their official duties; on a worldwide, perpetual, irrevocable, royalty-free basis to BMJ Publishing Group Ltd ("BMJ") its licensees and where the relevant Journal is co-owned by BMJ to the co-owners of the Journal, to publish the Work in this journal and any other BMJ products and to exploit all rights, as set out in our licence.*

*The Submitting Author accepts and understands that any supply made under these terms is made by BMJ to the Submitting Author unless you are acting as an employee on behalf of your employer or a postgraduate student of an affiliated institution which is paying any applicable article publishing charge ("APC") for Open Access articles. Where the Submitting Author wishes to make the Work available on an Open Access basis (and intends to pay the relevant APC), the terms of reuse of such Open Access shall be governed by a Creative Commons licence – details of these licences and which Creative Commons licence will apply to this Work are set out in our licence referred to above.*

*Other than as permitted in any relevant BMJ Author's Self Archiving Policies, I confirm this Work has not been accepted for publication elsewhere, is not being considered for publication elsewhere and does not duplicate material already published. I confirm all authors consent to publication of this Work and authorise the granting of this licence.*

For peer review only

# Title

UTAH: Using Telemedicine to improve early medical Abortion at Home – a protocol for a randomised controlled trial comparing face-to-face with telephone consultations for women seeking early medical abortion.


# Authors

John J Reynolds-Wright[1,2] email: jjrw@doctors.org.uk
John Norrie[3] email: j.norrie@ed.ac.uk
Sharon Cameron[1,2] email: Sharon.cameron@ed.ac.uk

# Affiliations

[1]MRC Centre for Reproductive Health, University of Edinburgh, Edinburgh, UK
[2]Chalmers Centre, NHS Lothian, Edinburgh, UK
[3]Edinburgh Clinical Trial Unit, University of Edinburgh, Edinburgh, UK

# Corresponding Author:

John J Reynolds-Wright
MRC Centre for Reproductive Health
The Queen's Medical Research Institute
Edinburgh BioQuarter
47 Little France Crescent
Edinburgh
EH16 4TJ
email: jjrw@doctors.org.uk


Word Count: 2732

Key Words: Gynaecology, Reproductive medicine, Community Gynaecology

For peer review only

# Abstract

### Introduction

Early medical abortion (EMA) is a two-stage process of terminating pregnancy using oral mifepristone (a progesterone-receptor antagonist) followed usually 1-2 days later by sublingual, vaginal or buccal misoprostol (a prostaglandin analogue). There are no published randomised controlled trials on the use of telemedicine for early medical abortion (EMA). Our proposed research will determine if telephone consultations for EMA (the commonest method of abortion in the UK) is non-inferior to standard face-to-face consultations with regards to efficacy of EMA.

### Methods and analysis

This study will be conducted as a randomised controlled trial (RCT). The recruitment target is 1222 participants.
The primary outcome is success of EMA (complete abortion rate). This will be determined, based upon a negative low-sensitivity urine pregnancy test result (2 weeks after misoprostol use), and absence of surgical intervention or diagnosis of ongoing pregnancy (within 6 weeks of misoprostol).
Secondary outcomes include: total time spent at a clinic appointment to receive EMA, self-reported preparedness for EMA, level of satisfaction with consultation and effective contraception uptake compared to when women attend for a face-to-face consultation.
The main analysis will be a modified intention to treat analysis. This will include all randomised women (with a viable pregnancy) using EMA, and follow-up for the main outcome. The study initiated on 13th January 2020 and is anticipated to finish in late 2021.

### Ethics and dissemination

Ethical approval was given by the South East Scotland NHS Research Ethics Committee, reference: 19/SS/0111. Results will be published in peer-reviewed journals, presented at clinical and academic meetings, and shared with participants via the clinic website.

### Registration details

UTAH was registered with clinicaltrials.gov on 25th October 2019. Unique identifier: NCT04139382.


### Strengths and limitations of this study

- This is the first study devised as a randomised-controlled trial to evaluate the safety and efficacy of telemedicine in early medical abortion.

For peer review only

- The study is design is aligned with the CONSORT, SPIRIT and Medical Abortion Reporting of Efficacy (MARE) recommendations in order to maximize the rigor and quality of the trial.
- The large sample size will allow a statistically and clinically meaningful analysis of the results.
- Due to the legal requirement for administration of mifepristone in in a clinical facility, participants in the telemedicine arm of the study will attend clinic to receive medications.
- The primary outcome relies on participant report of low sensitivity urine pregnancy test result.

For peer review only

## Introduction

We plan a trial comparing telephone consultations for women (see Box 1) requesting early medical abortion (EMA - under 10 weeks pregnant) to regular face-to-face consultations. In Scotland in 2018, 7 out of 10 women having an abortion chose EMA (1). In many settings, including Scotland, the clinic visit for a consultation to discuss a request for EMA is lengthy. Women can struggle with time off work or childcare for daytime appointments. There is evidence from observational studies that telephone consultations for EMA may be a safe and acceptable alternative(2-6). In our study, women seeking EMA will be randomised to face-to-face (standard care) or a planned telephone consultation (in advance of the clinic visit). We will determine the success of EMA in both groups, women's satisfaction with the consultation and possible advantages and disadvantages of the telephone consultation. If the study shows that success of EMA is maintained with a telephone consultation and that this model is acceptable to women, then this may change EMA provision throughout Scotland and other countries.

**Box 1: Language regarding gender**

Within this protocol we use the terms woman and women's health. However, it is important to acknowledge that there are people other than women for whom it is necessary to access women's health and reproductive services in order to maintain their gynaecological health and reproductive wellbeing. Gynaecological, sexual and reproductive health services must be appropriate, inclusive and sensitive to the needs of those individuals whose gender identity does not align with the sex they were assigned at birth.

Adapted from the RCOG/FSRH/BSACP Abortion care in Covid-19 guidance (22)

### Background

Abortion care is common, with approximately 1 in 3 women experiencing abortion in their lifetime worldwide(7). Each year approximately 200,000 abortions are performed in the UK and around 13,000 of these are in Scotland(8). 99% of abortions are delivered by the NHS in Scotland (1), compared to England and Wales, where 70% are delivered outside the NHS by the independent sector (8). Furthermore, Scotland has higher uptake of medical methods of abortion compared to England and Wales. In Scotland in 2017, 80% of all abortions were conducted in early pregnancy (under 10 weeks) and over 90% of these were EMA (1). The World Health Organization recommend that women can reliably self-manage much of EMA with support from a clinician (9).

In Scotland, women who choose an EMA, typically make a single visit to a clinic for a consultation and for assessment of gestation, receipt of mifepristone (to be administered in clinic, as per UK legal requirements) and misoprostol (to self-administer at home), receipt of contraception and instructions on how to self-assess the success of the abortion (using a self-performed urinary pregnancy test) (10,11,12). This clinic visit can last 2-3 hours; much of which may be time spent in the waiting room. Moreover, a significant proportion of consultation time is standard

For peer review only - http://bmjopen.bmj.com/site/about/guidelines.xhtml

history taking and information giving and could be delivered via the telephone, an app or video call rather than face-to-face.
Telephone consultations could add flexibility for women (e.g. consultation in the evening), reduce footfall in clinics (shorter time spent in clinic) and allow for more flexible staff working (office working, evening working, etc). There is observational evidence from other countries where abortion is legal to support use of telemedicine (including telephone consultations) for assessment of EMA (2-6). It is also possible that the consultation in advance of a clinic visit (for confirmation of decision, ultrasound and to collect medications) could mean that the subsequent clinical encounter is shorter, with possible efficiencies for the service, such as more effective use of medical staffing. It may also be easier to discuss and provide ongoing contraception at this encounter as women will have had time to digest the information about EMA provided at the telephone consultation. There is some observational evidence that telephone counselling may be associated with higher uptake of post abortion contraception (13). This could translate into fewer subsequent unintended pregnancies for women. Around 2400 abortions take place in NHS Lothian annually (1) and most women (80%) women attend a community abortion service at Chalmers Centre. In 2018, over 70% of abortions in this service were EMA (14).
We wish to determine if telephone consultations for assessment of women who are potentially eligible for EMA are non-inferior to face-to-face consultations (in terms of successful outcome of EMA). We designed a study of a telephone consultation assessment service via a randomised controlled trial (RCT). This has not been conducted before. This will be conducted within the framework of the 1967 Abortion Act (15). This RCT will provide robust data to support future service development nationally. Telephone consultations may make abortion services more accessible for women (especially those with work or child care commitments and vulnerable women). There is the possibility that services will become more efficient and so be able to provide cover for 'remote' services at other sites or health boards. Women could have an ultrasound for gestational dating and any other tests locally, but with consultations delivered by telephone. The aims of this study are in line with current Scottish Government policy on realistic medicine and on greater use of telemedicine services (16). Our Patient and Public Involvement group have helped develop this protocol and will continue to be involved throughout.

## Rationale for Study

There are no published randomised controlled trials on use of telemedicine for EMA. The existing evidence base is observational and exists outside of the NHS healthcare framework and outside of the UK medicolegal framework. There are only 5 studies that report outcomes of EMA that have been conducted in settings where abortion is legal (USA, Canada, Australia) with much heterogeneity (2-6). Our proposed research has the potential to confirm that telemedicine for EMA (the commonest method of abortion in Scotland) is non-inferior to standard face-to-face consultation with regards to efficacy.

There are no common outcome sets for abortion care research. An initiative to develop this is currently underway but is not scheduled to be complete until late 2021(17). In the absence of a common outcome set, we selected efficacy of EMA as

For peer review only

the primary outcome as recommended by the Medical Abortion Reporting of Efficacy (MARE) guidance(18). We hypothesise that inferior consultations could have an impact on women's ability to self-manage EMA and so wish to determine whether telemedicine consultations are inferior to face-to-face consultations with regard to efficacy. This RCT will gather robust data regarding success of EMA, duration of consultations, women's satisfaction with the consultation and uptake of effective contraception post abortion. These outcomes were identified from previous studies and developed in partnership with patients and public. The questionnaires used to collect this information were developed by the research team and reviewed and amended by our patient and public involvement group (Abortion Rights Edinburgh). The questionnaires were piloted with a group of patients and refined prior to the formal launch of the study.

These findings can be used to inform service development and abortion care strategy at a national level in Scotland and elsewhere, potentially impacting on the delivery of abortion care in many legal and restricted settings.

The primary research question is 'Is a telemedicine consultation for EMA non-inferior to a face-to-face consultation?' The secondary research questions are: 'How do the consultations compare with regard to patient satisfaction, time taken, and uptake of effective methods of contraception?'

For peer review only

# Methods and analysis

## Study Design

This study will be conducted as a randomised controlled trial (RCT) to compare telemedicine, specifically by telephone, with face-to-face consultations for women considering EMA at home.

### Primary Objective

To determine if EMA conducted following a telephone consultation is as effective (complete abortion rate) as following face-to-face consultation

### Secondary Objectives

To determine if a telephone consultation for EMA is associated with less total time spent at a clinic appointment to receive EMA, preparedness for EMA, level of satisfaction with consultation, rate of unscheduled contact with care, and effective contraception uptake compared to when women attend for a face-to-face consultation.

### Primary Endpoint

Success of EMA as defined by complete abortion rate without surgical intervention. This will be determined, based upon self-reported negative low-sensitivity urine pregnancy test result (2 weeks after misoprostol). The clinical database will be reviewed at 6 weeks post misoprostol to confirm final outcome of pregnancy and any admission or surgical intervention.

### Secondary Endpoints

- Women's reported 'preparedness' for EMA as assessed by pre-abortion questionnaire, when they collect their pack of medications.
- Satisfaction with consultation type as assessed by post-consultation questionnaire, conducted by telephone at 2 weeks.
- Uptake of effective contraception after EMA as assessed by case note review. Proportion of patients that are ineligible for EMA following ultrasound scan
- Total time spent in clinic (both telephone and face-to-face groups) and time taken for telephone consultation
- Unscheduled contact with abortion service or hospital within 6 weeks of EMA for concern related to EMA.

## Study Population

A total of 1222 participants randomised to receive telephone consultation (n=611) or face-to-face (n=611).
The success of EMA (primary outcome – complete abortion without surgical intervention) is assumed as 97%, based upon review of success rates in our regional database, as success rates in the literature are reported variably (usually between

For peer review only - http://bmjopen.bmj.com/site/about/guidelines.xhtml

95 and 99%). The recruitment target has been calculated using a binary outcome non-inferiority calculator with 90% power, one-sided 5% level of significance, 3% non-inferiority limit, 1:1 allocation and 10% compensation for loss to follow up (19). This will give us an adequately powered sample that will show statistical significance in efficacy findings.

The NHS Lothian abortion service cares for approximately 2400 women each year and of those 70% would be eligible to participate in the study. Over 18 months we should achieve adequate recruitment even if 50% of potential participants decline to participate and so should be feasible to complete within the projected timeframe.

### Inclusion Criteria

- Self-reported last menstrual period (LMP) less than 10 weeks on day of appointment
- Self-referral to Lothian Abortion Referral Service (LARS)
- Aged 16 or over at the time of procedure
- Preference for EMA
- Ability to give informed consent

### Exclusion Criteria

- Requires interpreter
- Patient preference for surgical method of abortion

## Identifying Participants

The administrative staff of LARS will collect the routine demographic information, basic obstetric history and contact details from women who self- refer for abortion (by telephone) and give them the next available date for the clinic so that participants in both study arms will receive an ultrasound scan, blood tests and sexual health screening as per usual care.

For women who meet the inclusion criteria, administrative staff will then read a short script about the study. If women express interest in participating, then permission will be sought for the research doctor or nurse to contact them by telephone at a convenient time to woman to discuss study participation. Interested women will also be directed to the clinic website where they can read the Participant Information Sheet (PIS) and consent form in advance of the call from the research doctor or nurse.

## Consent

Consent will be obtained from participants by the research doctor or nurse verbally over the telephone using a standard form. The participant will then be randomised to receive either a telephone consultation or a face-to-face consultation. When participants arrive in clinic, they will be asked to sign an affirmation that they continue to consent in the project.

The Participant Information Sheet and Consent Form are available as Supplementary File Appendix 1.

Randomisation lists will be generated by the Edinburgh Clinical Trials Unit (ECTU) and randomisation is performed by research staff using REDCap (Research Electronic Data Capture) software hosted at University of Edinburgh (20,21).

For peer review only

## Withdrawal of Participants

Participants are free to withdraw from the study at any point or a participant can be withdrawn by the Investigator should they no longer meet the inclusion/exclusion criteria for the study. If withdrawal occurs, the primary reason for withdrawal will be documented in the participant's case report form, if possible. The participant will have the option of withdrawal from all aspects of the trial but continued use of data collected up to that point. To safeguard rights, the minimum personally identifiable information possible will be collected.

## Study Assessments

| Assessment | When | Administered by | Description | Study Arm |
|---|---|---|---|---|
| Consultation duration | During telephone consultation/face-to-face consultation | Research Doctor or Nurse | Duration of face-to-face/telephone assessment consultation plus time spent in clinic on day of attendance. | Both arms |
| Questionnaire 1 | At the abortion clinic, following consultation prior to commencing abortion | Research Doctor or Nurse | A researcher-administered questionnaire identifying how prepared participant feels for EMA, how satisfied they were with consultation, and plans for contraception following EMA. Demographic information will also be collected at this point. | Both arms |
| Questionnaire 2 | Over the telephone/online/by post 14-20 days following EMA | Research Nurse or Doctor or self. | A researcher administered questionnaire to assess outcome of abortion by self-reported LSUPT outcome, satisfaction with whole abortion process and contraceptive outcome. | Both arms |

*Table 1. Study Assessments*

Study assessments are detailed in Table 1. There is no long term follow up. Participants are followed up at two weeks post abortion only. Questionnaire 2 will be primarily conducted by telephone, however, if women are not able to answer the telephone we will offer the option to receive the questionnaire via email or post to maximise response rate. Some study outcomes will be retrieved from routinely-collected clinical data and not included in this table.
Questionnaires 1 and 2 are available as Supplementary Files Appendix 2 and Appendix 3.

For peer review only

## Data Collection

Baseline characteristics: demographics, reproductive history and gestational age (based on ultrasound) will be collected on all participants.

Consultation time: duration of telephone consultation (minutes) and duration of face-to-face clinic consultation (minutes), total time spent in clinic on day of attendance for assessment (minutes)

Participant preparedness questionnaire: At clinic on first attendance – research nurse or doctor administered questionnaire to assess how prepared they feel.

Participant acceptability questionnaire: At two weeks post abortion - research nurse administered telephone questionnaire using validated questions on acceptability of consultation. Alternatively, this can be self-completed online or a paper postal questionnaire (if participant is unavailable via telephone or expresses a strong preference for this mode).

Outcome of abortion: self-reported outcome of routine low sensitivity urine pregnancy test at 2 weeks, plus review of clinical database at 6 weeks to confirm final outcome of pregnancy.

Unscheduled contact (in person or telephone) with abortion service or hospital for concern related to EMA within 6 weeks (clinical records review at six weeks)

## Data Management

### Personal Data

The following personal data will be collected as part of the research, we note that this data is already routinely collected in clinical practice as part of clinical history:
Name
Post code (in order to convert to the Scottish Index of Multiple Deprivation)
Weight, height, BMI
Previous pregnancy history
Physical personal data will be stored by the research team at Chalmers Centre, NHS Lothian, in the research office, behind a locked door that requires an ID badge to access and inside a locked cabinet in the room.

Study participants are assigned a numerical code to act as their identifier and is used when recording responses on paper and electronic data capture forms.

Electronic personal data will be kept on an NHS Lothian shared drive in password protected files. Passwords will be kept by research team and a hard copy with the locked physical data.
Identifiable personal data will be stored for a maximum of 5 years. Totally de-identified data will be retained for 10 years in total.

Data will be shared with colleagues at the University of Edinburgh Clinical Trials Unit (ECTU) who will assist with database management and statistical support.

For peer review only

### Transfer of Data

Data collected or generated by the study (including personal data) will not be transferred to any external individuals or organisations outside of the Sponsoring organisations.

### Data Controller

The University of Edinburgh and NHS Lothian are joint data controllers.

### Data Breaches

Any data breaches will be reported to the University of Edinburgh and NHS Lothian Data Protection Officers who will onward report to the relevant authority according to the appropriate timelines if required.

## Statistics and Data Analysis

### Proposed analyses

Statistical analysis will be conducted in partnership with the Edinburgh Clinical Trials Unit, University of Edinburgh.

Descriptive statistics will be used to characterize participants and assess comparability of the two groups at baseline.

For the primary outcome (efficacy of EMA), the main analysis will be a modified intention to treat analysis. This will include all randomised women, undergoing medical abortion, with a viable pregnancy (i.e. not ectopic, molar), and follow up for the main outcome recorded within 6 weeks of the abortion treatment.

A sensitivity analysis will be performed on an intention-to-treat population consisting of all randomised women having had medical abortion with viable pregnancy. We will impute the outcome for women lost to follow up.

Secondary outcomes will be analysed using appropriate tests depending upon the normality of the data: for normally distributed data we plan to use independent and paired t-tests, for non-normally distributed and categorical data we plan to use a combination of Mann-Whitney, Kruskal-Wallis and chi-squared testing. Results will be considered statistically significant if P-value $<0.05$.

No interim analysis is planned.

## Patient and Public Involvement

We consulted Abortion Rights Edinburgh, a local abortion and women's rights activism group. They kindly provided feedback on the trial rationale, study design

For peer review only

and study protocol prior to submission for ethical approval. They have agreed to disseminate the trial findings to their membership and via their networks.

## Ethics and dissemination

### Ethical approval

Ethical approval has been granted by South East Scotland NHS Research Ethics Committee on 28th October 2019, reference: 19/SS/0111.

### Dissemination plan

Results will be published in peer-reviewed journals, and as presentations at national and international meetings. All data will be reported in full. Participants will be able to access a summary of the trial results via the clinic website. Abortion Rights Edinburgh will disseminate to their membership and networks. The findings are likely to influence national and international guidance on best practice provision of abortion care.

## Study Status

The study opened to recruitment on 13th January 2020 and is temporarily paused due to service, legal and clinical guidance changes during covid-19, meaning that all patients are currently receiving telemedicine care(22). The status of telemedicine care in under legal review in Scotland (and England and Wales) and the outcome of this is expected later in 2021 and will determine whether recruitment can recommence.

## Administrative Details

UTAH was registered with clinicaltrials.gov on 25th October 2019. Unique identifier: NCT04139382.
UTAH is jointly sponsored by the University of Edinburgh (UK) and NHS Lothian (UK) via the ACCORD partnership and assigned the identifier AC19076. Protocol Version: 1.0; Date 18th September 2019.
The sponsor reviewed the study design and gave research and development approval to the trial. They are not involved in the collection, management, analysis or interpretation of the data, nor will they be involved in any report writing.

The research team are: John Reynolds-Wright (Clinical Research Fellow), Anne Johnstone (Clinical Research Nurse), Karen McCabe (Clinical Research Midwife), Claire Nicol (Lead Nurse, Abortion Service) and Sharon Cameron (Principle and Chief Investigator).

For peer review only

Authors' contributions: JJRW and SC equally contributed to the design of the protocol. JN contributed to the statistical analysis and sampling sections of the protocol.

Funding statement: This work was supported by the Edinburgh Family Planning Trust grant number EFPT/2019/UTAH. The project was conducted from the MRC Centre for Reproductive Health, supported by the Medical Research Council (grant MR/N022556/1).

Competing interests statement: Professor John Norrie was Deputy Chair of the National Institute of Health Research (NIHR) Health Technology Assessment (HTA) General Funding Committee (2016-2019) and is currently Chair of the Medical Research Council (MRC)/NIHR Efficacy and Mechanisms Evaluation (EME) Funding Committee.

Professor Sharon Cameron has no competing interests to declare.

Dr John Reynolds-Wright has no competing interests to declare.

For peer review only

## References

1.Information Services Division (ISD) Scotland, NHS National Services Scotland. Termination of Pregnancy Statistics: year ending December 2018. http://www.isdscotland.org/Health-Topics/Sexual-Health/Abortions/ (accessed 07 March 2020)

2. Hyland P, Raymond EG, Chong E. A direct-to-patient telemedicine abortion service in Australia: Retrospective analysis of the first 18 months. Aust N Z J Obstet Gynaecol 2018;58(3):335-340.

3. Grossman D, Grindlay K. Safety of medical abortion provided through telemedicine compared with in person. Obstet Gynaecol 2017;130(4):778-782.

4. Grossman D, Grindlay K, Buchacker T, Lane K, Blanchard K. Effectiveness and acceptability of medical abortion provided through telemedicine. Obstet Gynecol 2011;118:296–303

5. Wiebe ER. Use of telemedicine for providing medical abortion. International journal of gynaecology and obstetrics: the official organ of the International Federation of Gynaecology and Obstetrics. 2014;124(2):177-8

6. Kohn JE, Snow JL, Simons HR, Seymour JW, Thompson T, Grossman D. Medication Abortion Provided Through Telemedicine in Four U.S. States Obstetrics & Gynecology: August 2019 - Volume 134 - Issue 2 - p 343-350 doi: 10.1097/AOG.0000000000003357

7. Singh S, Remez L, Sedgh G, Kwok L, Onda T. Abortion Worldwide 2017: Uneven Progress and Unequal Access [Internet]. Vol. 2019. New York: Guttmacher Institute; 2018. Available from: https://www.guttmacher.org/report/abortion-worldwide-2017

8. Abortion Statistics for England and Wales 2018. https://www.gov.uk/government/statistics/abortion-statistics-for-england-and-wales-2018 (accessed 07 March 2020)

9. World Health Organization. Health worker roles in providing safe abortion care and post-abortion contraception. http://www.who.int/reproductivehealth/publications/unsafe_abortion/abortion-task-shifting/en/. (Accessed 07 March 2020)

10. Scottish Government. Chief Medical Officer Directorate. Abortion - Improvement to existing services - Approval for Misoprostol to be taken at home. https://www.sehd.scot.nhs.uk/cmo/CMO(2017)14.pdf (accessed 07 March 2020).

11. Cameron ST, Glasier A, Johnstone A, Dewart H, Campbell A. Can women determine the outcome of medical termination themselves? Contraception. 2015;91(1):6-11

For peer review only

12. Millar S, Cameron ST. Comparison of two low-sensitivity urine pregnancy tests for confirming the success of early medical abortion. BMJ Sex Reprod Health 2018;44(1):54-57

13. Lohr PA, Aiken ARA, Forsyth T, Trussell J. Telephone or integrated contraception counselling before abortion: impact on method choice and receipt. BMJ Sex Reprod Health. 2018; 44(2):114-121

14. Finch R, McGeechan K, Johnstone A, Cameron S. Impact of the introduction of 'home' as a legal setting for self-administration of misoprostol for early medical abortions: a prospective observational cohort study. BMJ Sex Reprod Health 2019 Aug 17. pii: bmjsrh-2018-200278. doi: 10.1136/bmjsrh-2018-200278. [Epub ahead of print]

15. UK Government. The Abortion Act 1967. Accessed via: http://www.legislation.gov.uk/ukpga/1967/87 on 07 March 2020.

16. Calderwood C. Realising Realistic Medicine: Chief Medical Officer for Scotland annual report 2015-2016. Scottish Government. 27 February 2017. ISBN: 9781786526731. Accessible via: https://www.gov.scot/publications/chief-medical-officer-scotland-annual-report-2015-16-realising-realistic-9781786526731/pages/8/

17. Whitehouse KC, Kim CR, Ganatra B, Duffy JMN, Blum J, Brahmi D, et al. Standardizing abortion research outcomes (STAR): a protocol for developing, disseminating and implementing a core outcome set for medical and surgical abortion. Contraception [Internet]. 2017/01/05 ed. 2017;95(5):437–41. Available from: https://www.ncbi.nlm.nih.gov/pubmed/28065833

18. Creinin MD, Chen MJ. Medical abortion reporting of efficacy: the MARE guidelines. Contraception. 2016;

19. Sealed Envelope Ltd. 2012. Power calculator for binary outcome non-inferiority trial. [Online] Available from: https://www.sealedenvelope.com/power/binary-noninferior/ [Accessed Sat Mar 07 2020]

20. PA Harris, R Taylor, R Thielke, J Payne, N Gonzalez, JG. Conde, Research electronic data capture (REDCap) – A metadata-driven methodology and workflow process for providing translational research informatics support, *J Biomed Inform. 2009 Apr;42(2):377-81.*

21. PA Harris, R Taylor, BL Minor, V Elliott, M Fernandez, L O'Neal, L McLeod, G Delacqua, F Delacqua, J Kirby, SN Duda, REDCap Consortium, The REDCap consortium: Building an international community of software partners, *J Biomed Inform. 2019 May 9 [doi: 10.1016/j.jbi.2019.103208]*

*22.* Royal College of Obstetricians and Gynaecologists. Coronavirus (COVID-19) infection and abortion care: Information for healthcare professionals [Internet]. London; 2020 Apr [cited 2020 Jun 4]. Available from: https://bsacp.org.uk/wp-content/uploads/2020/05/2020-04-09-coronavirus-covid-19-infection-and-abortion-care.pdf

For peer review only

Academic and Clinical Central Office for Research and Development

UTAH
PISCF 28 OCT 2019 v3.0
IRAS Project ID: 264265

# Participant Information Sheet

## UTAH: Using Telemedicine to improve early medical Abortion at Home

**You are invited to take part in a research study. To help you decide whether or not to take part, it is important for you to understand why the research is being done and what it will involve. Please take time to read the following information carefully. Talk to others about the study if you wish. Contact us if there is anything that is not clear, or if you would like more information. Take time to decide whether or not you wish to take part.**

## What is the purpose of the study?

You are being asked to take part in a study comparing the use of telephone consultations to face-to-face consultations in the delivery of care for women thinking about having an early medical abortion at home.

Face-to-face consultations are currently the 'standard care' in NHS Lothian, however, in many services across the world, for example in parts of Canada and Australia, telephone consultations are used routinely and safely. Telephone consultations are also used by some UK services for women living at a distance from a clinic and can be convenient for women.

There has never been a study comparing telephone consultations to face-to-face consultations. The purpose of this study is to fill in that gap and find out if telephone consultations are as good as a face to face consultation for determining if medical abortion at home is suitable and for providing the information that a woman wishes about this. We also want to know if telephone consultations take more or less time (or the same time) as a face to face consultation and if women prefer them to a face-to-face consultation.

## Why have I been invited to take part?

Women who intend to have a medical method of abortion at home and whose last menstrual period is less than 10 weeks prior to their appointment are eligible to take part. A total of 1222 women are being recruited to the study, with half (611) receiving the standard face-to-face consultation and half (611) receiving a telephone consultation before a clinic visit.

## Do I have to take part?

No, it is up to you to decide whether or not to take part. If you do decide to take part you will be given this information sheet to keep and be asked to sign a consent form. If you decide to take part you are still free to withdraw at any time and without giving a reason. Deciding not to take part or withdrawing from the study will not affect the healthcare that you receive, or your legal rights.

For peer review only - http://bmjopen.bmj.com/site/about/guidelines.xhtml

## What will happen if I take part?

When you call the clinic to make an appointment to discuss abortion, the receptionist on the telephone will give you a date and time to the attend the clinic, and will also invite you to take part in the study if you meet the eligibility criteria. If you agree to take part, you will receive a call back at a convenient time from a member of the research team, either a doctor or a nurse, who will explain the study in more detail and obtain your consent to participating over the phone.

You will then be randomly allocated by a computer to one group of the study, (you will know which group you are in): Telephone Group or Face-to-Face Group.

Telephone group: you will proceed to having your consultation on the telephone either immediately or at a later time, whichever is convenient for you. You will still attend the clinic at the date and time given to you in order to have all of the usual routine tests which include an ultrasound scan (to confirm how many weeks pregnant you are), have a blood test taken (for infections and for blood group) and a swab (that you take yourself) for infection. At this visit you would also be provided with the medical treatment to end the pregnancy, assuming that this is what you wish and that this is still appropriate for you based on the ultrasound scan of how many weeks pregnant you are at the clinic. Your chosen method of contraception can also be provided for you at this visit. If you change your mind, prefer a different method or based on the stage of the pregnancy a different method is indicated, you will be able to see a doctor or nurse in the clinic to discuss and plan this.

Face-to-face group: You will attend the clinic at the date and time given to you in order to have all of the usual routine tests (ultrasound scan, blood test and a swab). You will have the usual consultation with a doctor or nurse and proceed to treatment as usual. Your chosen method of contraception can also be provided for you at this visit. Participating in this arm of the study only involves completion of a short survey as described below.

For both groups, all of the tests and medical abortion treatments are the same.
For women in both groups a research nurse will ask you about your experience of the telephone or face to face consultation by a short telephone call interview (10 mins) two weeks after the treatment. If you prefer you can also choose to answer the same questions about your experience by completing a paper or online survey (whichever you prefer).

For peer review only

## What are the possible benefits of taking part?

Direct benefits: If you are in the telephone group, you may spend less total time in the clinic, although it is also possible that you may spend the same time or longer than if you were in the face to face consultation group– this is one of the outcomes that the study is investigating. With the telephone consultation it is possible that you may have the consultation at a time and place that suits you better, minimising disruption to your daily life.

Indirect benefits: Your participation will help us to know how the two consultation options compare in terms of the outcome of the medical abortion, women's acceptability of the consultations and how long the different consultations take. This will help inform us as to whether telephone consultations should be introduced as an option for women seeking a medical abortion at home in Scotland

For peer review only - http://bmjopen.bmj.com/site/about/guidelines.xhtml

## What are the possible disadvantages of taking part?

If you are randomised to the telephone group, you will not be able to see the person you are speaking to and this may affect the satisfaction you have with the process. The content of the conversation and the verbal and written information that you receive will be the same.
If you are randomised to the face-to-face group, you do not face any additional risk or disadvantage compared to not participating in the study, as this is the usual standard care at the clinic. You will only have the inconvenience of completing the questionnaires that form part of the study, but these are brief and can be completed by the research nurse at a short telephone call (10 minutes).

## What if there are any problems?

If you have a concern about any aspect of this study please contact [Removed for Publication], Research Nurse on [Telephone Number removed for publication], who will do their best to answer your questions.
In the unlikely event that something goes wrong and you are harmed during the research and this is due to someone's negligence then you may have grounds for a legal action for compensation against NHS Lothian but you may have to pay your legal costs. The normal National Health Service complaints mechanisms will still be available to you (if appropriate).

## What will happen if I don't want to carry on with the study

You are free to withdraw from the study at any time, without this affecting your care at the clinic whatsoever. If you do withdraw from the study, any non-identifiable data already collected will be retained.

## What happens when the study is finished?

When the study ends, identifiable data will be retained for 5 years in line with NHS Lothian Policy. Your data will be stored on an NHS Lothian Computer/Server. With your permission, some of your anonymised data will be kept for up to 10 years and may be used in future studies, but this information will not be directly linked to you and other researchers will not be able to identify you from it.

## Will my taking part be kept confidential?

All information which is collected about you during the course of the research will be kept strictly confidential. Any published information will not contain any identifiable information. If during the study, you disclose information that means either you or another person (adult or child) may be at risk of harm, we will need to break confidentiality and inform the clinical team and any additional appropriate agencies as per the clinic's policy. If this happens, we will inform you at the time. For details on what data will be held about you and who will hold and store this information please refer to the Data Protection Information Sheet.

## What will happen to the results of the study?

The results of the study will be published in a medical journal and presented at an international conference about reproductive health and contraception. Women who take

For peer review only - http://bmjopen.bmj.com/site/about/guidelines.xhtml

part in the study will not be able to be identified in any publication. If you wish, we can supply a summary of the findings to you via an email or postal address.

## Who is organising and funding the research?

This study has been organised by the MRC Centre for Reproductive Health, University of Edinburgh and the Chalmers Centre for Sexual and Reproductive Health, NHS Lothian. Additionally, The Edinburgh Clinical Trials Unit (part of University of Edinburgh) will be providing database support, statistical and general trial management support. The study has been sponsored by ACCORD, a partnership between the University of Edinburgh and NHS Lothian.

The study is being funded by Edinburgh Family Planning Trust.

## Who has reviewed the study?

The study proposal has been reviewed by NHS Lothian Research and Development.

The public have been involved in the development of this study, via review of the protocol and study documents by members of a community action group that support women in Edinburgh who receive abortion care.

All research in the NHS is looked at by an independent group of people called a Research Ethics Committee. A favourable ethical opinion has been obtained from South East Scotland REC 01. NHS Management Approval has also been given.

## Researcher Contact Details

If you have any further questions about the study please contact [deleted for publication], Research Nurse on [telephone number] or email on: [email address]

## Independent Contact Details

If you would like to discuss this study with someone independent of the study please contact [deleted for publication], Consultant Gynaecologist on [email address]

## Complaints

If you wish to make a complaint about the study please contact:

[details removed for publication]

You can also do this through the NHS Complaints Procedure:
Patient Experience Team,
[details removed for publication]

For peer review only - http://bmjopen.bmj.com/site/about/guidelines.xhtml

Academic and Clinical Central Office for Research and Development

UTAH
PISCF 28 OCT 2019 v3.0
IRAS Project ID 264265

**Participant ID:** **Participant name:**

# RECORD OF VERBAL CONSENT

## UTAH: Using Telemedicine to improve early medical Abortion at Home

Please **initial** box

1. I confirm that the participant has read and understood the information sheet (DD MMM YYYY and Version Number) and the Data Protection Information Sheet (DD MMM YYYY and Version Number) for the above study. They have had the opportunity to consider the information, ask questions and have had these questions answered satisfactorily. ☐
2. The participant understands that their participation is voluntary and that they are free to withdraw at any time, without giving any reason and without their medical care and/or legal rights being affected. ☐
3. The participant gives permission for the research team to access their medical records for the purposes of this research study. ☐
4. The participant understands that relevant sections of their medical notes and data collected during the study may be looked at by individuals from the Sponsor (University of Edinburgh and NHS Lothian), from regulatory authorities or from the NHS organisation where it is relevant to their taking part in this research. They give permission for these individuals to have access to their data and/or medical records. ☐
5. The participant gives permission for their personal information (including name, address, date of birth, telephone number and consent form) to be passed to the University of Edinburgh and Edinburgh Clinical Trials Unit for administration of the study. ☐
6. The participant understands that data collected about them during the study may be converted to anonymised data. ☐
7. The participant agrees to their anonymised data being used in future ethically approved studies. Yes ☐ No ☐
8. The participant agrees to take part in the above study. ☐

For peer review only

| Name of Person Receiving Consent | Date | Signature |
|---|---|---|

**On the day of clinic attendance – please sign to confirm ongoing consent to participate and agreement with the above statements**

| Name of Participant | Date | Signature |
|---|---|---|

1x original – into Site File; 1x copy – to Participant; 1x copy – into medical record

For peer review only - http://bmjopen.bmj.com/site/about/guidelines.xhtml

UTAH – Questionnaire 1 STUDY NUMBER: __________

Name of interviewer__________________________________________

Date of interview (dd/mm/yy)____________________________________

We would be grateful if you would spend some time filling out this questionnaire. It should take you about 10 minutes. The questionnaire asks about your experience of your recent consultation for medical abortion and your plans for contraception.

**Please CIRCLE your response.**

1. What kind of consultation did you receive?
    a. Face-to-face
    b. Telephone
    c. Telephone at first but then another consultation in clinic (NOTE: this means having a new consultation with a doctor, not just meeting the nurse to collect your medicines)
2. How acceptable did you find having your consultation this way?
    a. Very acceptable
    b. Somewhat acceptable
    c. Neutral
    d. Somewhat unacceptable
    e. Very unacceptable
3. How acceptable have you found the whole process so far?
    a. Very acceptable
    b. Somewhat acceptable
    c. Neutral
    d. Somewhat unacceptable
    e. Very unacceptable
4. Now that you have had your consultation, how well prepared do you feel?
    a. Very prepared
    b. Somewhat prepared
    c. Neutral
    d. Somewhat unprepared
    e. Very unprepared

5. How satisfied with the consultation you had?
    a. Very satisfied
    b. Somewhat satisfied
    c. Neutral
    d. Somewhat unsatisfied
    e. Very unsatisfied
6. Do you feel that all of your questions were answered by the consultation?
    a. Yes
    b. I have some questions still
    c. No, none of my questions were answered
7. What did you think of duration of the consultation?
    a. Much too long
    b. A bit longer than I wanted
    c. Just right
    d. A bit shorter than I wanted
    e. Much too short
8. What time of day was your consultation?
    a. Morning (08.00 – 12.00)
    b. Afternoon (12.00-17.00)
    c. Evening (17.00-20.00)
9. How convenient was the time of day of your consultation?
    a. Very convenient
    b. Somewhat convenient
    c. Neutral
    d. Somewhat inconvenient
    e. Very inconvenient
10. What method of contraception (if any) are you planning to start following your abortion treatment?
    a. Combined hormonal contraceptive pill / patch or ring
    b. Progestogen only pill (mini pill)
    c. Male condom
    d. Contraceptive injection' jag' (Depo Provera or Sayana)
    e. Implant (Nexplanon)
    f. Copper Coil/intra-uterine device (IUD)

g. Intrauterine system (Mirena or Jaydess)
h. Female condom
i. Cap/diaphragm
j. Partner has been sterilised (vasectomy)
k. I have been sterilised
l. I am currently pregnant
m. Other method of protection-**please write here what this is**
   ______________________________________________
n. I am not planning to use any method of contraception

UTAH – Questionnaire 2 STUDY NUMBER: __________

We would be grateful if you would spend some time filling out this questionnaire. It should take you about 10 minutes. The questionnaire asks about your experience of your recent consultation for medical abortion, the result of your pregnancy test and if you chose a method of contraception.

**Please CIRCLE responses.**

1. What kind of consultation did you receive?
    a. Face-to-face
    b. Telephone
    c. Telephone at first but then another consultation in clinic (NOTE: this means having a new consultation with a doctor, not just meeting the nurse to collect your medicines)
2. Looking back, how acceptable did you find having your consultation this way?
    a. Very acceptable
    b. Somewhat acceptable
    c. Neutral
    d. Somewhat unacceptable
    e. Very unacceptable
3. Looking back, what did you think of duration of the consultation?
    a. Much too long
    b. A bit longer than I wanted
    c. Just right
    d. A bit shorter than I wanted
    e. Much too short
4. Looking back, how well prepared were you?
    a. Very prepared
    b. Somewhat prepared
    c. Neutral
    d. Somewhat unprepared
    e. Very unprepared
5. If you had a good friend who was thinking about having an abortion knowing what you know now, would you recommend the same type of consultation?
    a. Yes
    b. No
    c. Not sure

UTAH – Questionnaire 2 STUDY NUMBER: __________

6. Did you start the method of contraception you left clinic with?
    a. Yes
    b. No – I have since chosen a different method
    c. No – I have not started but I am waiting for a coil/IUD/IUS
    d. No – I did not leave with a method
7. If you were to design the perfect service, which of the following would be important to you:

| | Very Unimportant | Somewhat Unimportant | Neutral | Somewhat Important | Very Important |
|---|---|---|---|---|---|
| Evening face-to-face clinic | | | | | |
| Evening telephone consultation | | | | | |
| Skype or video consultation | | | | | |
| A mobile phone app to send/receive information in advance | | | | | |
| Online booking | | | | | |
| Medication posted to me | | | | | |
| Medication that could collect from a local pharmacy | | | | | |
| Able to get the treatment from my GP | | | | | |

# Reporting checklist for protocol of a clinical trial.

Based on the SPIRIT guidelines.

## Instructions to authors

Complete this checklist by entering the page numbers from your manuscript where readers will find each of the items listed below.

Your article may not currently address all the items on the checklist. Please modify your text to include the missing information. If you are certain that an item does not apply, please write "n/a" and provide a short explanation.

Upload your completed checklist as an extra file when you submit to a journal.

In your methods section, say that you used the SPIRITreporting guidelines, and cite them as:

Chan A-W, Tetzlaff JM, Gøtzsche PC, Altman DG, Mann H, Berlin J, Dickersin K, Hróbjartsson A, Schulz KF, Parulekar WR, Krleža-Jerić K, Laupacis A, Moher D. SPIRIT 2013 Explanation and Elaboration: Guidance for protocols of clinical trials. BMJ. 2013;346:e7586

| | | Reporting Item | Page Number |
|---|---|---|---|
| **Administrative information** | | | |
| Title | #1 | Descriptive title identifying the study design, population, interventions, and, if applicable, trial acronym | 1 |
| Trial registration | #2a | Trial identifier and registry name. If not yet registered, name of intended registry | 2 |
| Trial registration: data set | #2b | All items from the World Health Organization Trial Registration Data Set | 2 |
| Protocol version | #3 | Date and version identifier | 9 |
| Funding | #4 | Sources and types of financial, material, and other support | 9 |
| Roles and responsibilities: | #5a | Names, affiliations, and roles of protocol contributors | 9 |

For peer review only

| | | | |
|---|---|---|---|
| contributorship | | | |
| Roles and responsibilities: sponsor contact information | #5b | Name and contact information for the trial sponsor | 9 |
| Roles and responsibilities: sponsor and funder | #5c | Role of study sponsor and funders, if any, in study design; collection, management, analysis, and interpretation of data; writing of the report; and the decision to submit the report for publication, including whether they will have ultimate authority over any of these activities | 9 |
| Roles and responsibilities: committees | #5d | Composition, roles, and responsibilities of the coordinating centre, steering committee, endpoint adjudication committee, data management team, and other individuals or groups overseeing the trial, if applicable (see Item 21a for data monitoring committee) | n/a |
| **Introduction** | | | |
| Background and rationale | #6a | Description of research question and justification for undertaking the trial, including summary of relevant studies (published and unpublished) examining benefits and harms for each intervention | 3 |
| Background and rationale: choice of comparators | #6b | Explanation for choice of comparators | 4 |
| Objectives | #7 | Specific objectives or hypotheses | 5 |
| Trial design | #8 | Description of trial design including type of trial (eg, parallel group, crossover, factorial, single group), allocation ratio, and framework (eg, superiority, equivalence, non-inferiority, exploratory) | 5 |
| **Methods: Participants, interventions, and outcomes** | | | |

For peer review only

| | | | |
|---|---|---|---|
| Study setting | #9 | Description of study settings (eg, community clinic, academic hospital) and list of countries where data will be collected. Reference to where list of study sites can be obtained | 5 |
| Eligibility criteria | #10 | Inclusion and exclusion criteria for participants. If applicable, eligibility criteria for study centres and individuals who will perform the interventions (eg, surgeons, psychotherapists) | 5,6 |
| Interventions: description | #11a | Interventions for each group with sufficient detail to allow replication, including how and when they will be administered | 5 |
| Interventions: modifications | #11b | Criteria for discontinuing or modifying allocated interventions for a given trial participant (eg, drug dose change in response to harms, participant request, or improving / worsening disease) | n/a |
| Interventions: adherance | #11c | Strategies to improve adherence to intervention protocols, and any procedures for monitoring adherence (eg, drug tablet return; laboratory tests) | n/a |
| Interventions: concomitant care | #11d | Relevant concomitant care and interventions that are permitted or prohibited during the trial | n/a |
| Outcomes | #12 | Primary, secondary, and other outcomes, including the specific measurement variable (eg, systolic blood pressure), analysis metric (eg, change from baseline, final value, time to event), method of aggregation (eg, median, proportion), and time point for each outcome. Explanation of the clinical relevance of chosen efficacy and harm outcomes is strongly recommended | 5 |
| Participant timeline | #13 | Time schedule of enrolment, interventions (including any run-ins and washouts), assessments, and visits for participants. A schematic diagram is highly recommended (see Figure) | 6/7 |
| Sample size | #14 | Estimated number of participants needed to achieve study objectives and how it was determined, including | 5 |

For peer review only

| | | | |
|---|---|---|---|
| | | clinical and statistical assumptions supporting any sample size calculations | |
| Recruitment | #15 | Strategies for achieving adequate participant enrolment to reach target sample size | 6 |
| **Methods: Assignment of interventions (for controlled trials)** | | | |
| Allocation: sequence generation | #16a | Method of generating the allocation sequence (eg, computer-generated random numbers), and list of any factors for stratification. To reduce predictability of a random sequence, details of any planned restriction (eg, blocking) should be provided in a separate document that is unavailable to those who enrol participants or assign interventions | 6 |
| Allocation concealment mechanism | #16b | Mechanism of implementing the allocation sequence (eg, central telephone; sequentially numbered, opaque, sealed envelopes), describing any steps to conceal the sequence until interventions are assigned | n/a |
| Allocation: implementation | #16c | Who will generate the allocation sequence, who will enrol participants, and who will assign participants to interventions | 6 |
| Blinding (masking) | #17a | Who will be blinded after assignment to interventions (eg, trial participants, care providers, outcome assessors, data analysts), and how | n/a |
| Blinding (masking): emergency unblinding | #17b | If blinded, circumstances under which unblinding is permissible, and procedure for revealing a participant's allocated intervention during the trial | n/a |
| **Methods: Data collection, management, and analysis** | | | |
| Data collection plan | #18a | Plans for assessment and collection of outcome, | 7 |

For peer review only

| | | | |
|---|---|---|---|
| | | baseline, and other trial data, including any related processes to promote data quality (eg, duplicate measurements, training of assessors) and a description of study instruments (eg, questionnaires, laboratory tests) along with their reliability and validity, if known. Reference to where data collection forms can be found, if not in the protocol | |
| Data collection plan: retention | #18b | Plans to promote participant retention and complete follow-up, including list of any outcome data to be collected for participants who discontinue or deviate from intervention protocols | 7 |
| Data management | #19 | Plans for data entry, coding, security, and storage, including any related processes to promote data quality (eg, double data entry; range checks for data values). Reference to where details of data management procedures can be found, if not in the protocol | 7 |
| Statistics: outcomes | #20a | Statistical methods for analysing primary and secondary outcomes. Reference to where other details of the statistical analysis plan can be found, if not in the protocol | 8 |
| Statistics: additional analyses | #20b | Methods for any additional analyses (eg, subgroup and adjusted analyses) | 8 |
| Statistics: analysis population and missing data | #20c | Definition of analysis population relating to protocol non-adherence (eg, as randomised analysis), and any statistical methods to handle missing data (eg, multiple imputation) | 8 |
| **Methods: Monitoring** | | | |
| Data monitoring: formal committee | #21a | Composition of data monitoring committee (DMC); summary of its role and reporting structure; statement of whether it is independent from the sponsor and competing interests; and reference to where further details about its charter can be found, if not in the protocol. Alternatively, an explanation of why a DMC is not needed | n/a |
| Data monitoring: | #21b | Description of any interim analyses and stopping | 8 |

For peer review only

| | | | |
|---|---|---|---|
| interim analysis | | guidelines, including who will have access to these interim results and make the final decision to terminate the trial | |
| Harms | #22 | Plans for collecting, assessing, reporting, and managing solicited and spontaneously reported adverse events and other unintended effects of trial interventions or trial conduct | n/a |
| Auditing | #23 | Frequency and procedures for auditing trial conduct, if any, and whether the process will be independent from investigators and the sponsor | n/a |
| **Ethics and dissemination** | | | |
| Research ethics approval | #24 | Plans for seeking research ethics committee / institutional review board (REC / IRB) approval | 9 |
| Protocol amendments | #25 | Plans for communicating important protocol modifications (eg, changes to eligibility criteria, outcomes, analyses) to relevant parties (eg, investigators, REC / IRBs, trial participants, trial registries, journals, regulators) | 9 |
| Consent or assent | #26a | Who will obtain informed consent or assent from potential trial participants or authorised surrogates, and how (see Item 32) | 6 |
| Consent or assent: ancillary studies | #26b | Additional consent provisions for collection and use of participant data and biological specimens in ancillary studies, if applicable | n/a |
| Confidentiality | #27 | How personal information about potential and enrolled participants will be collected, shared, and maintained in order to protect confidentiality before, during, and after the trial | 8 |
| Declaration of interests | #28 | Financial and other competing interests for principal investigators for the overall trial and each study site | 9 |
| Data access | #29 | Statement of who will have access to the final trial dataset, and disclosure of contractual agreements that limit such access for investigators | n/a |

For peer review only

| | | | |
|---|---|---|---|
| Ancillary and post trial care | #30 | Provisions, if any, for ancillary and post-trial care, and for compensation to those who suffer harm from trial participation | n/a |
| Dissemination policy: trial results | #31a | Plans for investigators and sponsor to communicate trial results to participants, healthcare professionals, the public, and other relevant groups (eg, via publication, reporting in results databases, or other data sharing arrangements), including any publication restrictions | 9 |
| Dissemination policy: authorship | #31b | Authorship eligibility guidelines and any intended use of professional writers | n/a |
| Dissemination policy: reproducible research | #31c | Plans, if any, for granting public access to the full protocol, participant-level dataset, and statistical code | n/a |

**Appendices**

| | | | |
|---|---|---|---|
| Informed consent materials | #32 | Model consent form and other related documentation given to participants and authorised surrogates | appendix 1 |
| Biological specimens | #33 | Plans for collection, laboratory evaluation, and storage of biological specimens for genetic or molecular analysis in the current trial and for future use in ancillary studies, if applicable | n/a |

The SPIRIT checklist is distributed under the terms of the Creative Commons Attribution License CC-BY-ND 3.0. This checklist was completed on 04. November 2020 using https://www.goodreports.org/, a tool made by the EQUATOR Network in collaboration with Penelope.ai

For peer review only
